# The Antiaging Potential of Dietary Plant-Based Polyphenols: A Review on Their Role in Cellular Senescence Modulation

**DOI:** 10.3390/nu17101716

**Published:** 2025-05-19

**Authors:** Matteo Centonze, Emanuela Aloisio Caruso, Valentina De Nunzio, Miriam Cofano, Ilenia Saponara, Giuliano Pinto, Maria Notarnicola

**Affiliations:** Laboratory of Nutritional Biochemistry, National Institute of Gastroenterology IRCCS “Saverio de Bellis”, 70013 Castellana Grotte, Italy; matteo.centonze@irccsdebellis.it (M.C.); emanuela.caruso@irccsdebellis.it (E.A.C.); valentina.denunzio@irccsdebellis.it (V.D.N.); miriam.cofano@irccsdebellis.it (M.C.); ilenia.saponara@irccsdebellis.it (I.S.); giuliano.pinto@irccsdebellis.it (G.P.)

**Keywords:** senescence, polyphenols, nutraceuticals, senolytics, senotherapeutics

## Abstract

Aging is a complex biological process characterized by a progressive decline in physiological functions and an increased risk of chronic diseases. A key mechanism of this process is cellular senescence, the permanent arrest of the cell cycle in response to stress or damage, which contributes to the accumulation of dysfunctional cells in tissues. Recent research has highlighted the role of polyphenols, bioactive compounds present in numerous plant-based foods, in positively modulating these processes. Polyphenols exert antioxidant effects, regulate gene expression and improve mitochondrial function, helping to delay cellular aging and prevent age-related diseases. In addition, some polyphenols exhibit senolytic properties, selectively eliminating senescent cells and promoting tissue regeneration. This review summarizes the current evidence on the effects of polyphenols on aging and cellular senescence, exploring the underlying molecular mechanisms and discussing their potential in nutritional strategies aimed at promoting healthy aging.

## 1. Introduction

Human life expectancy has increased globally due to significant advances in healthcare and medicine. However, aging remains a major risk factor for a wide range of chronic diseases, including cardiovascular and metabolic disorders, neurodegenerative conditions, and various cancers—together contributing substantially to global morbidity, mortality, and healthcare costs [1]. Although pharmacological strategies have been developed to target individual age-related conditions, their overall impact on multimorbidity and late-life health outcomes has been limited. Aging is increasingly recognized as a systemic biological process driven by cellular and molecular mechanisms, including the accumulation of senescent cells. Once considered solely a tumor-suppressive response, cellular senescence is now known to play a pivotal role in the development and progression of age-related pathologies. In recent years, several molecules with senotherapeutic action have been synthesized, but great emphasis is also being placed on dietary components that we normally consume and which, in several studies, have shown the ability to prevent senescent cells from accumulating in tissues.

### 1.1. Cell Senescence

During aging, senescent cells (SCs) accumulate across various tissues and organs, and this alters their physiologic function, leading to age-related diseases [2]. The phenomenon of cellular senescence was first described in the 1960s by Hayflick and colleagues, who observed that human diploid fibroblasts exhibited a finite number of divisions under in vitro conditions—a limitation later attributed to telomere shortening [3]. Since then, extensive research has shown that senescence can be triggered by a wide range of stressors such as DNA damage, oxidative stress, epigenetic modifications, oncogene activation, mitochondrial dysfunction, and even environmental factors such as high-fat diets.

SCs are defined by an irreversible arrest of the cell cycle, primarily mediated through the p53–p21 and p16–Rb signaling pathways, while retaining metabolic activity [4,5].

Additionally, SCs exhibit elevated levels of lipofuscin and senescence-associated β-galactosidase (SA-β-gal), and present the formation of DNA segments with chromatin alterations reinforcing senescence (DNA-SCARS), all of which contribute to the stable maintenance of the senescent state [6].

In addition to permanent cell cycle arrest, SCs activate various damage response signaling pathways, including p38 mitogen-activated protein kinases (p38 MAPK) and nuclear factor kappa-light-chain-enhancer of activated B cells (NF-κB). These pathways contribute to the acquisition of resistance to apoptosis and the formation of senescence-specific DNA structures such as senescence-associated DNA damage foci (SDF) and senescence-associated heterochromatin foci (SAHF), both reflective of altered chromatin architecture [7].

One of the most defining features of SCs is the development of a pro-inflammatory and proteolytic secretory profile known as the senescence-associated secretory phenotype (SASP). The SASP includes a diverse set of bioactive molecules—cytokines, chemokines, growth factors, proteases, and lipids—that can drive or exacerbate numerous age-related diseases, including cancer [8]. This secretory phenotype is considered a key link between cellular senescence and the chronic, low-grade inflammation observed with aging, often referred to as ‘inflammaging’ [8].

Senescence is not inherently detrimental; in fact, it can have beneficial roles under specific physiological conditions. Transient or acute senescence supports tissue remodeling, wound healing, embryogenesis, and even tumor suppression. It also contributes to maintaining tissue homeostasis and enhancing insulin secretion by pancreatic β-cells during aging [9]. However, when senescence becomes chronic, the persistent presence of SCs and sustained SASP signaling can be harmful. Accumulated SCs promote tissue dysfunction and contribute to the pathogenesis of a wide range of age-related diseases, such as neurodegenerative disorders, cancer, arthritis, vision loss, atherosclerosis, and type 2 diabetes mellitus [10].

**Figure 1:**
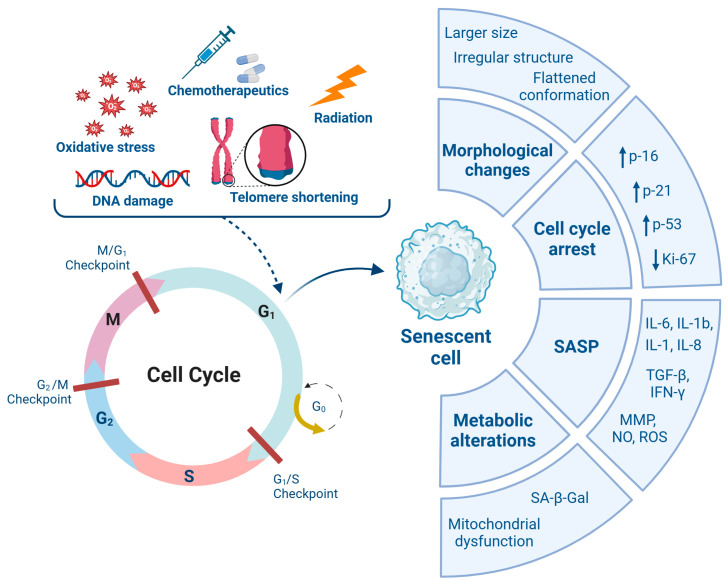
Induction triggers and hallmarks of cellular senescence. Various cellular stressors can trigger irreversible cell cycle arrest, culminating in the establishment of cellular senescence. Senescent cells exhibit hallmark features, including morphological changes, metabolic reprogramming, and molecular alterations. Abbreviations: SASP: Senescence-Associated Secretory Phenotype; TGF-β: Transforming Growth Factor beta; IFN-γ: Interferon Gamma; MMP: Matrix Metalloproteinase; NO: Nitric Oxide; ROS: Reactive Oxygen Species; SA-β-Gal: Senescence-Associated Beta-Galactosidase.

Cancer, as a prototypical aging-associated disease, shares a complex relationship with senescence. While senescence initially acts as a barrier to tumorigenesis by halting the proliferation of damaged cells, the pro-tumorigenic factors released via the SASP can create a supportive environment for neighboring premalignant or malignant cells [11]. Furthermore, SASP components secreted by senescent stromal cells in the tumor microenvironment have been shown to reduce the effectiveness of anticancer therapies and contribute to tumor relapse and resistance [12,13]. Figure 1 summarizes the factors that can induce cellular senescence and the resulting phenotypic characteristics.

### 1.2. Senolytics and Senomorphics

SCs and SASP are promising targets for strategies aimed at enhancing both lifespan and healthspan. An increasing number of studies have demonstrated the therapeutic potential of selectively targeting SCs using either natural or synthetic compounds, collectively referred to as senotherapeutics. For instance, genetically eliminating p16-expressing SCs in mouse models of BubR1-deficiency-associated progeria and of chronic diseases has been shown to restore tissue function and significantly extend healthspan [14].

Senotherapeutics are broadly classified into two categories: senolytics and senomorphics. Senolytics are small molecules that selectively induce apoptosis in SCs. Most senolytics identified to date achieve this by targeting critical enzymes involved in prosurvival and antiapoptotic mechanisms, such as members of the BCL-2 protein family [15,16]. Senomorphics, on the other hand, are compounds that suppress the SASP without inducing cell death, thereby indirectly mitigating the effects of cellular senescence. These agents typically interfere with SASP-related signaling pathways—such as p38 MAPK, mTOR, PI3K/Akt, and JAK/STAT—as well as transcription factors like NF-κB, C/EBPβ, and STAT3. They may also neutralize the action of specific SASP components such as IL-1α, IL-6, and IL-8 using targeted antibodies [17].

While both senolytic and senomorphic therapies have demonstrated efficacy in vivo, they are not without limitations—most notably, dose-dependent side effects and potential toxicity. This has spurred interest in nutraceuticals—bioactive compounds derived from plants and foods—which may offer similar benefits with fewer adverse effects [5].

**Figure 2:**
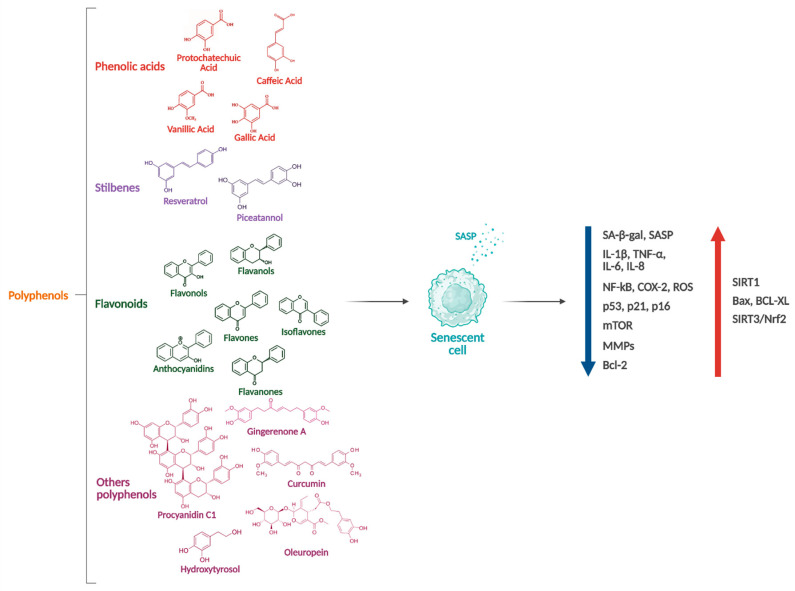
Polyphenol-mediated modulation of cellular senescence. Polyphenols exert modulatory effects on cellular senescence by downregulating the expression of senescence-associated markers, pro-inflammatory cytokines, and cell cycle regulators, while inhibiting key pro-senescent signaling pathways. They simultaneously enhance the activation of cytoprotective pathways and influence the expression of apoptotic regulators. Abbreviations: SA-β-Gal: Senescence-Associated Beta-Galactosidase; SASP: Senescence-Associated Secretory Phenotype; TNF-α: Tumor Necrosis Factor Alpha; NF-kB: Nuclear Factor Kappa-light-chain-enhancer of activated B cells; COX-2: Cyclooxygenase-2; ROS: Reactive Oxygen Species; mTOR: mammalian Target Of Rapamycin; MMPs: Matrix Metalloproteinases; Bcl: B Cell Lymphoma; SIRT: Sirtuin; Bax: Bcl-2 associated X protein; Nrf2: Nuclear factor erythroid 2-related factor 2.

Natural product-derived compounds have shown the ability to reduce the burden of senescence and exert anti-inflammatory effects in both in vitro and in vivo studies, including in animal models and humans, highlighting their promise as senotherapeutics. Among these, polyphenols—a diverse group of plant-derived compounds—have attracted significant attention for their ability to modulate cellular senescence (Figure 2).

## 2. Methods

The literature search was carried out using the electronic databases PubMed, Scopus, Web of Science, and Google Scholar. Search terms included combinations of keywords related to dietary polyphenols and cellular senescence, using Boolean operators (“AND”, “OR”) to refine the results. Only peer-review articles written in English were considered. Eligible studies included original research articles, systematic reviews, and meta-analyses including in vitro, in vivo (animal), and clinical research that evaluated the antiaging implications of dietary polyphenols and their effects on markers or mechanisms of cellular senescence. After removing duplicates, titles and abstracts were screened for relevance, followed by full-text analysis of eligible articles. Data were extracted and organized based on the type of polyphenol studied and main findings related to the modulation of cellular senescence.

## 3. Polyphenols

Structurally, polyphenols are characterized by one or more aromatic rings and two or more hydroxyl groups. They are widely found in vegetables, fruits, cereals, red wine, tea, and related derived foods and beverages [18]. Dietary polyphenols exhibit a range of beneficial biological activities, including antioxidant, anti-inflammatory, anticancer, antimicrobial, and neuroprotective effects [19]. Based on their chemical structure, dietary polyphenols can be classified into five major groups: phenolic acids (e.g., hydroxybenzoic and hydroxycinnamic acids), stilbenes, lignans, flavonoids (such as flavanols, isoflavones, anthocyanins, flavanones, flavones, and flavonols), and tannins [20]. Compounds within each subclass have shown potential health-promoting properties when regularly consumed as part of a balanced diet.

Growing evidence suggests that drugs developed from these compounds may be effective in treating a range of conditions, including neurological, cardiovascular, metabolic, cancer, and aging-related diseases [21]. In fact, dietary phytochemicals may extend lifespan by modulating metabolic pathways and cellular functions, in a manner similar to other antiaging interventions like regular physical activity, caloric restriction, and intermittent fasting [22]. Although naturally derived senolytics may be less potent than synthetic ones, their lower toxicity makes them attractive alternatives. This review explores the preventive and senotherapeutic potential of main polyphenols which have shown promise in preclinical studies involving both animal models and humans. The antisenescence effects, molecular mechanisms of the action of polyphenols, models, and experimental methods used in the above studies are summarized in Supplementary Table S1.

### 3.1. Phenolic Acids

Phenolic acids are a group of phenolic compounds that have an aromatic ring containing one or more hydroxyl groups. Based on the carbon skeleton, phenolic acids can be divided in two groups: hydroxycinnamic and hydroxybenzoic acids [23].

The basic structure of hydroxybenzoic acids is derived from benzoic acid, which consists of a benzene ring attached to a carboxylic acid group (-COOH), usually in position 1, and one or more hydroxyl groups (-OH) substituted onto the benzene ring at different positions. Representative compounds of hydroxybenzoic acids are gallic acid (GA), protocatechuic acid (PCA), vanillic acid, and syringic acids [24]. Hydroxycinnamic acids share the base structure of cinnamic acid with a three-carbon side chain (C6–C3) and one or more hydroxyl (-OH) or methoxy (-OCH₃) groups attached to the aromatic ring [25]. Among hydroxycinnamic acids include caffeic, ferulic, sinapic, coumaric, and chlorogenic acids. The treatment with GA of rat embryonic fibroblasts induced to senescence with H_2_O_2_ showed a reduction in β-galactosidase activity and a significant decrease of inflammatory cytokines and oxidative stress markers [26]. Werner syndrome (WS) is a premature aging syndrome. The treatment of WS human mesenchymal stem cells (hMSCs) with GA has an effect on delaying cellular replicative senescence. Moreover, the treatment of senescent hMSCs with GA showed a downregulation of senescent markers SA-β-gal, p21 and p16, an extended telomere length, and a reduction of Reactive oxygen species (ROS) and cell apoptosis [27]. *C. elegans* treated with PCA isolated from *V. peregrina* shows a dose-dependent increase in lifespan and in tolerance against osmotic, heat shock and oxidative stress [28]. PCA has an antioxidant and a senescence-attenuating effect on LPS-treated human dermal fibroblasts (HDFs). PCA reduces intracellular ROS induced by LPS and the number of β-gal-positive cells, regulating COL1A1 and MMP1 gene expression [29]. Vanillic acid increases stress resistance, reduces protein aggregation, improves motility, and extends the lifespan of *C. elegans* by nearly 50% [30].

D-Galactose (D-gal) can induce rat brain aging by increasing oxidative stress, leading to cellular senescence, memory deficits, and neuronal apoptosis by an overexpression of apoptotic proteins Bcl-2, Bax and caspase 3, and this effect can be attenuated by caffeic acid [31].

Caffeic, sinapic, and rosmarinic acids induce collagen synthesis in senescent HDFs and in co-culture of human keratinocytes (HaCaT) and HDF, inhibiting MMP-1 and IL-6 [32].

### 3.2. Stilbenes

Stilbenes are a class of bioactive phytochemicals characterized by a common structural motif consisting of two aromatic rings connected by a 2-carbon ethylene bridge, forming a compact structure with a central double bond. Among the numerous stilbenes identified in plants, only a limited number are commonly found in the human diet. The most extensively studied dietary stilbenes include trans-resveratrol (3,5,4′-trihydroxystilbene) and piceatannol, both of which have been associated with a variety of health-promoting effects due to their antioxidant, anti-inflammatory, and potential antiaging properties [33].

Resveratrol (RES) is a naturally occurring phytoalexin synthesized by various plant species in response to environmental stress, mechanical injury, ultraviolet (UV) radiation, and pathogenic attacks, particularly fungal infections such as those caused by *Botrytis cinerea*. Among dietary sources, red wine represents the most significant contributor of resveratrol and has garnered widespread scientific interest for its potential to promote healthy aging, as demonstrated across multiple animal models [34]. RES exhibits potent senomorphic properties, modulating key pathways involved in cellular senescence. Notably, it inhibits the NF-κB pathway, a central regulator of the SASP, while simultaneously activating nuclear factor erythroid 2–related factor 2 (Nrf2), a known negative regulator of NF-κB [35,36]. The importance of the role of the Nrf2 pathway in aging and the effects of its modulation by RES has been further confirmed by Franco et al. [37]. Mehrabi et al. [38] investigated the synergistic effects of resveratrol supplementation and high-intensity interval training (HIIT) on aging-related changes in the frontal lobe of aged rats. RES supplementation and HIIT have individual positive effects on the expression of SIRT4, SIRT5, SOD1, and SOD2 in the frontal lobe of aged rats and exhibits synergistic effects mitigating aging-related oxidative stress and improving brain health. In addition to its anti-inflammatory and antioxidant functions, RES influences the Sirtuin signaling pathway. Sirtuin 1 (SIRT1), a NAD⁺-dependent deacetylase, regulates various transcription factors associated with stress resistance and longevity. Under conditions of chronic inflammation and oxidative stress, SIRT1 activity is often diminished, whereas RES has been shown to restore and enhance SIRT1 expression and activity [39]. For instance, Liu et al. demonstrated that resveratrol significantly activates SIRT1, inhibits NF-κB signaling, and reverses the decline in intestinal stem cell populations [40]. The longevity-promoting effects of RES have been observed across a wide range of model organisms. It has been reported to extend the maximal lifespan of the budding yeast *Saccharomyces cerevisiae* [41], the nematode *C. elegans* [42], the fruit fly *Drosophila melanogaster* [43], and the honey bee *Apis mellifera* [44]. Furthermore, RES has demonstrated neuroprotective properties, as evidenced by its capacity to prevent cognitive impairment in Alzheimer’s disease (AD) models by reducing the production of pro-inflammatory cytokines [45], and to alleviate motor and cognitive deficits in a dose-dependent manner in a murine model of Parkinson’s disease (PD) [46]. RES has demonstrated promising antiaging effects in preclinical studies. In aged mice, dietary supplementation with RES led to a significant attenuation of age-related physiological decline, including reduced albuminuria, reduced cataract development, maintenance of bone mineral density, decreased vascular inflammation and endothelial apoptosis, improved aortic elasticity, and enhanced motor coordination [47]. Furthermore, when combined with human umbilical cord mesenchymal stem cell (hUC-MSC) transplantation, RES enhanced neurogenesis and improved learning and memory functions in AD mouse models [48]. Particular matter 2.5 (PM2.5) pollution is associated with senescence induction. In PM2.5-induced senescent dermal papilla stem cells, the treatment with RES decreased SA-β-gal expression, the mRNA levels of SASP proteins IL-7, IL-1α, IL-8, and CXCL1, and mRNA and protein expression of both p16 and p21 [49].

Notably, RES is capable of crossing the blood–brain barrier, where it may exert neuroprotective effects by inhibiting amyloid-β deposition and plaque formation [50]. It also promotes the viability and proliferation of hUC-MSCs in a dose-dependent manner, delaying cellular senescence and upregulating SIRT1 expression while downregulating pro-senescence markers such as p53 and p16 [51]. Despite encouraging preclinical evidence, the efficacy of RES in humans remains controversial. A 26-week clinical trial in 23 overweight elderly participants suggested potential benefits, including improved glucose metabolism, enhanced hippocampal connectivity, and better memory performance [52]. However, contrasting findings have emerged from a recent meta-analysis and a larger follow-up study involving 225 patients, both of which failed to demonstrate significant cognitive or memory improvements with RES supplementation [53]. These inconsistencies underscore the need for further large-scale, controlled clinical trials to elucidate RES’s true potential in age-related neurodegenerative conditions.

Piceatannol (PIC) is a stilbene structurally similar to RES, but has greater antioxidant power, greater bioavailability, and a longer half-life [54]. In acute (H_2_O_2_-induced) and chronic (replicative) senescent human mesenchymal stromal cells (MSCs), PIC seemed to have a senomorphic effect in the range of 0.01–1 μM and a senolytic effect at 10 μM. In H_2_O_2_-treated cells, PIC decreased the expression of RB1 and P21, while in chronic SCs, it decreased the expression of RB2, P16, and P27 [55]. In TIG-3 cells irradiated with X-ray and heavy-ion irradiation, PIC treatment suppressed X-ray-induced double strand breaks and reduced SA-β-gal staining intensity [56]. PIC may help improve age-related hearing loss in C57BL/6 mice by modulating the inflammatory response of inner ear hair cells and Caspase-4/11-mediated non-classical pyroptosis [57]. An extract of passion fruit seeds, which had been found to be rich in PIC, decreased UVB-induced senescence cellular oxidants and MMP-1 production in HaCaT cells, a human keratinocytes cell line [58]. PIC significantly extended the lifespan of *C. elegans* without altering the growth rate, worm size, and progeny production. PIC prolonged the lifespan of *C. elegans* through the insulin/IGF-1 signaling and sir-2.1-dependent pathway. It also enhanced resistance to heat and oxidative stress, while delaying age-associated declines in pharyngeal pumping and locomotor activity [59]. In an aging mouse model, PIC mitigates behavioral abnormalities and brain injury through activation of the Nrf2 pathway [60].

### 3.3. Flavonoids

**Figure 3:**
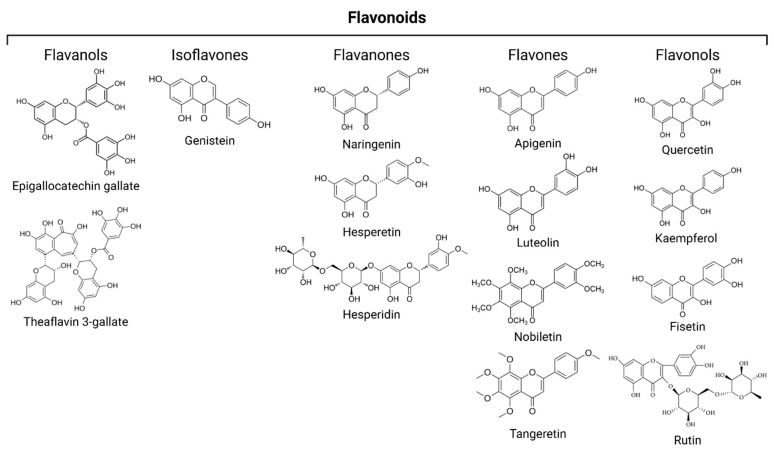
Five subclasses of flavonoids and structural formulas of representative compounds.

Structurally, flavonoids are polyphenolic phytochemicals prevalent in a wide range of fruits and vegetables, characterized by a common C6–C3–C6 skeleton comprising two aromatic rings (A and B) connected via a three-carbon bridge forming a heterocyclic ring [61]. A substantial body of preclinical research suggests that flavonoids may mitigate cellular senescence by influencing key signaling pathways implicated in aging and activating endogenous cytoprotective responses. Their ability to reduce systemic inflammation and oxidative stress, alongside the suppression of SCs and SASP, underscores their promise as potential senescence-targeting therapeutics. Notably, flavonoids are capable of traversing the blood–brain barrier, where they exert multifaceted effects—antioxidant, anti-inflammatory, and neuroprotective—on central nervous system cells, indicating therapeutic relevance for age-related neurodegenerative conditions. Epidemiological analyses have further linked higher dietary intake of flavonoid-rich foods to a decreased incidence of cardiovascular diseases and type 2 diabetes [62,63]. In comparison to synthetic compounds, these bioactive molecules generally exhibit a superior safety and tolerability profile, making them attractive candidates for long-term therapeutic applications. Flavonoids were divided in five subclasses: flavanols, isoflavones, flavanones, flavones, and flavonols (Figure 3).

#### 3.3.1. Flavanols

Flavanols, also known as flavan-3-ols, are a subclass of flavonoids characterized by a chemical structure that consist of two aromatic rings connected by a three-carbon bridge, which forms part of a six-membered, non-aromatic heterocyclic ring known as the C-ring. A hydroxyl group is attached at the third carbon of this ring, giving rise to the term flavan-3-ols. This structural framework contributes to their antioxidant properties and is commonly found in natural compounds such as catechin, epicatechin, and epigallocatechin. These compounds are abundant in foods like tea (especially green tea), cocoa, berries, and apples [64].

Epigallocatechin gallate (EGCG) is a flavanol and is one of the main polyphenolic constituents of green tea. Preclinical evidence suggests that EGCG exhibits promising senolytic and senomorphic effects through multi-faceted mechanisms. In senescent preadipocytes, EGCG can inhibit ROS, Cox2, and NF-kB and alleviate SASP expression (IL-6 and TNF-a) by activating the regulatory transcription factors Nrf2 and SIRT3 [65]. Moreover, in the same cell line, EGCG could protect against ROS production and DNA damage, induce apoptosis by inhibiting Bcl-2, and downregulate the activation of stress-induced PI3k/Akt/mTOR signaling [66]. In another study by Kumar et al., EGCG treatment could alleviate macrophage inflammation and senescence, and curb incidences of inflammatory disorders in the elderly [67].

EGCG modulates communication between senescent endothelial cells (ECs) and monocytes, attenuating age-related vascular inflammation. EGCG treatment reduced senescence-associated phenotypes in etoposide-induced senescent ECs, evidenced by decreased SA-β-Gal activity and reversal of etoposide-induced senescence markers such as CDKN1A, CDKN2A, CDKN2B, CXCL8, and IL6. When monocytes were co-cultured with EGCG-treated senescent ECs, they exhibited diminished pro-inflammatory responses compared to those co-cultured with untreated senescent ECs. Moreover, senescent ECs secreted more extracellular vesicles (EVs) than non-senescent ECs and these EVs enhanced lipopolysaccharide (LPS)-induced pro-inflammatory activation in monocytes. In contrast, EVs from EGCG-treated senescent ECs attenuated this response, maintaining monocyte activation at baseline levels. These findings suggest that EGCG exerts its antisenescent effects, in part, by modulating the ECs secretome—particularly through EV-mediated signaling—which in turn regulates monocyte inflammatory response [68]. SA-β-Gal staining of HDF cells treated with EGCG at early and late passages showed fewer positive cells in treated than controls. Moreover, p53 was shown to be significant reduced in EGCG treated cells. However, cell cycle analysis of HDFs with and without EGCG treatment showed that treated cells had a similar percentage of cell in S phase as control cells [69]. In H_2_O_2_-treated hMSCs, EGCG pretreatment reduces acetylated p53 and p21 protein levels, but loses its antioxidant effect in Nrf2-knockdown hMSCs, which indicates that EGCG prevents oxidative stress-induced cellular senescence through Nrf2 activation [70].

Theaflavins, the primary functional polyphenols responsible for the characteristic red coloration of black tea, possess a range of bioactivities, including free radical scavenging, antioxidant, antiapoptotic, and anti-inflammatory properties. Among these, theaflavin-3-gallate (TF2A) has demonstrated antisenescence effects, primarily through modulation of gene expression patterns associated with long non-coding RNAs (lncRNAs). TF2A has been shown to attenuate cellular senescence in multiple stem cell populations. Age-associated decline in hypothalamic neural stem cells (htNSCs) plays a pivotal role in driving systemic aging and age-related pathologies. Recent findings have identified a lncRNA, Hnscr, as a critical regulator of htNSC homeostasis. Hnscr is highly expressed in htNSCs of young mice but exhibits a pronounced decline in expression by middle age. Functional depletion of Hnscr is sufficient to induce cellular senescence within htNSCs and to precipitate aging-like phenotypes in vivo. Notably, TF2A has been shown to mimic Hnscr activity at the molecular level. In middle-aged mice, TF2A treatment attenuated htNSC senescence and concurrently ameliorated multiple aging-related phenotypes, underscoring its potential as a lncRNA-mimetic therapeutic for age-related neurodegeneration and systemic aging [71]. In bone-marrow-derived mesenchymal stem cells (BMSCs) of middle-aged mice, the expression of the lncRNA Gm31629 is significantly reduced compared to those of young counterparts. Functional ablation of Gm31629 is sufficient to induce cellular senescence in BMSCs, which in turn impairs bone regeneration in vivo. TF2A functions as a mimic of Gm31629, alleviating senescence in BMSCs and promoting bone regeneration [72].

#### 3.3.2. Isoflavones

Isoflavones are a class of polyphenolic compounds predominantly found in soy and other legumes, where they function as phytoestrogens. Structurally distinct from typical flavonoids, isoflavones feature the B-ring attached to the third position of the central heterocyclic C-ring, resulting in a characteristic 3-phenylchromen-4-one backbone. This unique arrangement underlies their diverse biological effects, including antioxidant, anti-inflammatory, and hormone-regulating properties [73].

Genistein, a naturally occurring isoflavone predominantly found in soy, has garnered attention for its role as a selective estrogen receptor modulator, exhibiting both estrogenic and antiestrogenic activities depending on the tissue context. Beyond its hormone-related actions, genistein has demonstrated promising antiaging properties at the cellular level, particularly in the vascular system.

In human vascular smooth muscle cells, genistein has been shown to attenuate cellular senescence by inhibiting the mechanistic target of rapamycin (mTOR), a central regulator of cell growth and metabolism that is commonly upregulated during aging. Concurrently, it promotes the activation of autophagy, a critical cellular process involved in the degradation and recycling of damaged organelles and proteins. This genistein-induced autophagy appears to be mediated via the liver kinase B1 (LKB1)–AMP-activated protein kinase (AMPK) signaling axis [74], which plays a vital role in cellular energy homeostasis and stress responses. Activation of the LKB1–AMPK pathway by genistein not only facilitates autophagic flux but also helps to mitigate oxidative stress and inflammation, both of which are hallmark features of vascular aging. Bone marrow mesenchymal stem cells from ovariectomized rats (OVX-BMMSCs) display multiple senescence-associated phenotypes, including elevated ROS levels, mitochondrial dysfunction, and premature cellular aging. Genistein treatment greatly ameliorated cellular senescence in OVX-BMMSCs. Network pharmacology analysis and molecular docking identified estrogen-related receptor alpha (ERRα) as a putative molecular target of genistein. Notably, knockdown of ERRα markedly abrogated genistein’s protective effects against senescence in OVX-BMMSCs, indicating that ERRα is essential for mediating its function. Moreover, genistein-induced mitochondrial biogenesis and mitophagy were significantly impaired upon ERRα knockdown. These findings suggest that genistein exerts its effects through the ERRα signaling axis to preserve mitochondrial integrity and function. In vivo, genistein supplementation in OVX rats effectively reduced trabecular bone loss and downregulated the expression of the senescence marker p16, while upregulating the expression of mitochondrial regulators such as sirtuin 3 (SIRT3) and peroxisome proliferator-activated receptor gamma coactivator 1-alpha (PGC-1α) in the trabecular bone region of the proximal tibia. Together, these findings provide compelling evidence that genistein ameliorates age-related decline in bone health by attenuating senescence through an ERRα-dependent mechanism that promotes mitochondrial biogenesis and mitophagy [75]. In addition to its protective roles in vascular smooth muscle cells and bone-marrow-derived stem cells, genistein has also been shown to exert significant antisenescent effects in endothelial cells. In human umbilical vein endothelial cells (HUVECs), genistein was found to inhibit senescence induced by oxidized low-density lipoprotein (ox-LDL), a known contributor to vascular aging and atherogenesis. This antisenescent effect was evidenced by a reduction in the expression of key senescence markers, including p16 and p21, as well as decreased activity of SA-β-gal [76].

#### 3.3.3. Flavanones

Flavanones are based on the 2,3-dihydro-2-phenylchromen-4-one skeleton. Chemically, they are characterized by a C6-C3-C6 structure with two phenyl rings (A and B rings) and a heterocyclic ring (C ring), and a saturation at the C2–C3 bond (unlike flavones, which have a double bond there).

Hesperidin and its aglycone, hesperetin, are prominent flavanones present in various fruits and vegetables, recognized for their antioxidant and anti-inflammatory properties. These compounds suppress the production of proinflammatory cytokines, thereby inhibiting the SASP. Hesperidin, in particular, may help combat pulmonary fibrosis by preventing lung fibroblast senescence, downregulating key senescence markers such as p53, p21, and p16, and reducing the number of β-galactosidase-positive cells [77]. In human senescent chondrocytes, hesperidin increases cellular antioxidant capacity and decreases the expression of SASP proinflammatory cytokines. mRNA levels of Cyclooxygenase-2 (COX-2), IL-1β, Tumor Necrosis Factor (TNF)-α, Matrix Metalloproteinase (MMP)-3, and MMP-9 were downregulated, while levels of IL-10, TIMP-1, and SOX9 were upregulated. Moreover, Foxo1, Foxo3, and Nrf2 signaling pathways were modulated by hesperidin. This suggests a beneficial role in the progression of osteoarthritis [78]. Similarly, hesperidin has been shown to protect against bone loss by inhibiting bone resorption, NF-kB activity, and improving bone mineral density in male senescent rats [79]. Hesperetin plays a pivotal role in lowering immune inflammation of the joints in rheumatoid arthritis by inhibiting cytokine production and c-Jun N-terminal kinase (JNK) activity in synovial fibroblasts [80].

In mice, Cisd2 protein levels decline with natural aging, and genetic studies have demonstrated that elevated Cisd2 expression extends both lifespan and healthspan. Hesperetin, a known activator of Cisd2, has been shown to increase Cisd2 levels in aged tissues when administered as a dietary supplement to naturally aged mice. This intervention significantly prolongs lifespan, mitigates age-related metabolic decline, reduces fat accumulation, enhances glucose homeostasis, and slows the aging of skeletal muscle [81]. In HEK001 cells, a human keratinocytes cell line derived from an old person, hesperetin improves mitochondrial function and protects against oxidative stress caused by ROS through the upregulation of CISD2, reduces UVB-induced cellular damage, and inhibits the expression of MMP-1, a key marker of UVB-induced photoaging in keratinocytes. Hesperidin also activates two transcription factors linked to longevity, FOXO3a and FOXM1, which help suppress the SASP [82].

Naringenin is the aglycone metabolite generated via enzymatic hydrolysis of naringin, the primary bitter flavonoid in citrus fruits [83]. Recent evidence demonstrates that naringenin enhances adult neurogenesis in the hippocampus of aged murine models, primarily through the suppression of pro-inflammatory signaling pathways, particularly via the downregulation of TNF-α-associated gene expression [84]. At the cellular level, naringenin has been shown to modulate redox homeostasis and mitochondrial bioenergetics, thereby mitigating H_2_O_2_-induced premature senescence in H9c2 myoblasts cell line, highlighting its capacity to preserve mitochondrial function under oxidative stress conditions [85]. Furthermore, in human dermal fibroblasts, naringenin promotes extracellular matrix biosynthesis and attenuates senescence phenotypes elicited by LPS and ROS exposure. These antisenescent and regenerative effects are mediated through the SIRT1-dependent inhibition of the NF-κB pathway, coupled with suppression of NADPH oxidase activity and MMP expression, indicating a coordinated regulatory mechanism underlying its dermoprotective and antiaging properties [86].

Naringenin has been shown to be effective in counteracting IL-1β-induced senescence in nucleus pulposus cells, probably through inhibition of IGFBP-3 expression [87].

After chronic administration for six months of naringenin to 6-month-old mice, an improvement of myocardium functionality can be observed, accompanied by increased expression of SIRT1 in myocardial tissue and a reduction of ROS production [88]. Supplementation with naringenin and activation of SIRT1 slow down the signs of brain aging in middle-aged mice, as a consequence of upregulated expression of Foxo3, Nrf2, Ho-1 (antioxidants), p16, IL-6, and IL-18 [89].

The administration of Naringenin ameliorated atherosclerotic lesion formation and vascular senescence in aged apoE^−/−^ mice and decreased ROS production and enhanced activity and expression of SIRT-1 and of its target genes FOXO3a and PGC1α [90].

#### 3.3.4. Flavones

Flavones are a class of flavonoids based on the backbone of 2-phenylchromen-4-one (2-phenyl-1-benzopyran-4-one) characterized by a benzene ring (A), a heterocyclic ring (C), and a second benzene ring (B), with a double bond between carbon 2 and 3 and a ketone group at position 4 on ring C [91].

Apigenin is a natural product belonging to the flavone subclass of flavonoids and is found in many fruits and vegetables, but parsley, celery, celeriac, and chamomile are the most common sources.

In bleomycin-induced senescent BJ cells, apigenin inhibits NF-κB signaling by preventing the phosphorylation and nuclear translocation of IκBζ, and consequently, suppressing SASP expression. These data were confirmed in vivo, where the oral administration of Apigenin reduced the elevated levels of SASP and IκBζ mRNA found in the kidneys of aged rats [92]. In senescent human fibroblasts, apigenin reduced IL-6 secretion and NF-κB activity stimulated by IL-1A through a reduction of IRAK1/IRAK4/p38MAPK phosphorylation, indirectly mitigating the aggressive phenotype of breast cancer cells stimulated by the SASP [93].

Apigenin has been shown to ameliorate AD-associated cognitive deficits in APP/PS1 double transgenic AD mice by reducing amyloid-β peptides accumulation, inhibiting oxidative stress, and restoring ERK/CREB/BDNF pathway [94]. Apigenin improves learning and memory in old mice and modulates transcriptomic signatures of inflammation/immune activation, in addition to reducing markers of senescence and inflammation in senescent primary human astrocytes [95]. Apigenin enhances the osteogenic differentiation potential of human bone-marrow-derived mesenchymal stem cells (hHBMCs), thereby facilitating new bone formation. Furthermore, it contributes to a reduction in cellular senescence and attenuates oxidative stress levels, suggesting its potential role in bone tissue regeneration and age-related bone loss mitigation [96].

Luteolin is a naturally occurring flavonoid that can be abundantly found in vegetables and fruits like pepper, celery, broccoli, and orange. It plays an important role in defending plants, for example against UV radiation by partially absorbing UVA and UVB radiation [97]. Luteolin possesses therapeutic potential against age-related hearing loss that is induced by oxidative stress. Treatment of H_2_O_2_-induced senescent House Ear Institute-Organ of Corti 1 cells (HEI-OC1) showed that luteolin attenuated senescent markers such as morphology alteration, SA-β-gal expression, DNA damage, and expression of p53 and p21. In this cell model, luteolin induced the expression of SIRT1 in a dose-dependent manner [98]. Luteolin mitigates TNF-α-induced inflammatory injury and senescence in nucleus pulposus cells by upregulating Sirtuin 6 expression and subsequently suppressing the activation of the downstream NF-κB signaling cascade [99]. Luteolin pretreatment significantly alleviated photoaging following UVA radiation of BALB/c mice and inhibited the expression of UVA-induced senescent factors P21, P16, and SASP in human fibroblasts both in vitro and in vivo [100].

Administration of Luteolin to D-gal-induced senescent rats significantly ameliorated oxidative stress, mitochondrial dysfunction, neuroinflammation, and cellular senescence in the hippocampus. Furthermore, luteolin treatment attenuated neuronal apoptosis and significantly upregulated SIRT1 mRNA expression levels in hippocampal tissue. These results suggest that luteolin exerts neuroprotective effects in an aging rat model, primarily through the modulation of SIRT1 signaling [101].

Nobiletin (5,6,7,8,3′,4′-hexamethoxyflavone) is a polymethoxyflavone isolated from citrus peel. Nobiletin has demonstrated promising antiaging and lifespan-extending properties in preclinical studies. For example, nobiletin can improve the survival and function of the human pancreatic islets isolated for transplantation through its anti-apoptotic, antioxidant, and insulinotropic properties [102]. Nobiletin has emerged as a promising therapeutic candidate for mitigating cognitive decline, oxidative stress, and tau hyperphosphorylation associated with aging and age-related neurodegenerative disorders, including AD. The senescence-accelerated mouse prone 8 (SAMP8) model, which exhibits early-onset learning and memory deficits alongside key pathological hallmarks of AD—such as elevated oxidative stress, tau hyperphosphorylation, and amyloid plaque deposition—was utilized to evaluate the neuroprotective effects of nobiletin.

Treatment with nobiletin significantly reversed impairments in recognition memory and context-dependent fear memory in SAMP8 mice. Moreover, nobiletin restored redox balance in the brain by normalizing the GSH/GSSG ratio and upregulating the activity of key antioxidant enzymes, including glutathione peroxidase and manganese-superoxide dismutase. These findings suggest that nobiletin exerts its neuroprotective effects through modulation of oxidative stress and enhancement of endogenous antioxidant defense systems [103]. Moreover, nobiletin has been shown to enhance autophagy and mitochondrial biogenesis through activation of the SIRT1/FOXO3a and PGC-1α pathways, respectively. In hepatic ischemia–reperfusion injury models, these mechanisms have been implicated in reversing tissue damage and improving liver function [104].

In a D-gala-induced C2C12 myoblast aging model, nobiletin delayed skeletal muscle aging, improving mitochondrial and autophagy function, inhibiting inflammation, increasing ATP production, clearing ROS, and reducing ROS production [105].

In the context of osteoarthritis (OA), nobiletin improved extracellular matrix protein synthesis in human chondrocytes and inhibited cartilage degradation by suppressing pro-inflammatory cytokine expression and blocking the PI3K/Akt and NF-κB pathways [106]. These findings underscore nobiletin’s potential as a senotherapeutic agent with additional disease-modifying capabilities. However, further preclinical validation and clinical investigation are warranted to fully elucidate its efficacy and translational relevance.

Tangeretin, a polymethoxylated flavone primarily derived from citrus peel, has emerged as a candidate senotherapeutic agent, with its bioactivity predominantly mediated via pro-apoptotic and anti-inflammatory mechanisms.

In *C. elegans*, tangeretin supplementation significantly prolonged mean lifespan, enhanced thermotolerance, and attenuated age-associated physiological decline. These effects were mechanistically linked to modulation of the insulin/insulin-like growth factor-1 signaling pathway. Specifically, tangeretin upregulated the transcription of daf-16, hsp-16.2, and hsp-16.49, and promoted nuclear translocation of the DAF-16 transcription factor, a critical effector of IIS-mediated longevity and stress resistance [107].

Neuroprotective effects have been demonstrated in vivo, wherein tangeretin mitigated cerebral ischemia-reperfusion injury in rats by suppressing NF-κB signaling, attenuating oxidative stress, and reducing the expression of proinflammatory cytokines [108]. These effects support its utility in attenuating ischemic brain injury and highlight its potential therapeutic application in cerebrovascular pathologies. Additionally, in microglia-mediated neuroinflammatory models, tangeretin exerted anti-inflammatory effects via downregulation of NF-κB and MAPK signaling cascades, concomitant with reduced expression of proinflammatory mediators such as TNF-α and IL-6 [109], suggesting potential utility in neurodegenerative disease contexts where chronic microglial activation is implicated.

#### 3.3.5. Flavonols

Flavonols, a prominent subclass of polyphenolic compounds, are widely distributed in dietary sources. Structurally, flavonols conform to the C6–C3–C6 backbone, characterized by two aromatic rings (A and B) linked via a three-carbon bridge forming a heterocyclic pyran ring (C-ring). This core includes a double bond between C2 and C3 and a ketone functional group at C4. The quantity and positional configuration of hydroxyl substituents on the flavonol scaffold critically influence their redox properties and broader bioactivity, including their capacity to modulate oxidative stress and cellular signaling pathways [110].

Quercetin, a micronutrient commonly found in the daily diet, is a natural flavonol known for its wide range of pharmacological properties. Quercetin exists not only in its free aglycone form but also in various conjugated forms, where glycosides or methyl ethers are bound to its hydroxyl groups [61].

In vitro experiments have demonstrated that quercetin acts as a geroprotective agent against premature and physiological human aging, as demonstrate by experiments on Werner Syndrome hHBMCs, Hutchinson–Gilford progeria syndrome, and physiological-aging hMSCs [111].

Quercetin is a proteosome activator that enhances lifespan and viability of HFL-1 primary human fibroblasts and promotes a rejuvenating effect when supplemented to already-senescent cells [112].

Adipose tissue is a site where many SCs accumulate. In H_2_O_2_-induced senescent preadipocytes and adipocytes treated with quercetin, a reduction in the number of SA-β-gal positive cells, ROS production, and inflammatory cytokines could be observed.

Quercetin can act as both senomorphic and senolytic. In doxorubicin-induced senescent WI-38 fibroblasts quercetin increased the endoplasmic reticulum stress by reducing autophagy, triggering SCs death. Moreover, conditioned medium from treated fibroblasts reduced osteosarcoma cell invasiveness [113].

In an obese and hypercholesterolemic mouse model, renal function and cortical oxygenation are impaired, and markers of senescence, such as p16, p19, p53, and SA-β-gal expression, are upregulated in renal tubular cells. Quercetin treatment reduced this condition and ameliorated renal fibrosis, increased renal cortical oxygenation, and decreased plasma creatinine levels [114].

Quercetin inhibited SASP factors expression and senescence phenotype in IL-1β-treated nucleus pulposus cells by binding to the Keap1-Nrf2 complex, thus suppressing the activation of the NF-κB pathway and ameliorating the intervertebral disk degeneration [115].

In vivo experiments have showed that quercetin is able to prolong lifespan and to increase stress resistance of organisms such as *Saccharomyces cerevisiae* [116] and *C. elegans* [117].

Despite its pleiotropic effects, quercetin alone demonstrates limited senolytic efficacy across heterogeneous SCs populations. Therefore, its combination with the tyrosine kinase inhibitor dasatinib (D + Q) has been shown to elicit synergistic senolytic activity. The D + Q combination exhibits enhanced senolytic activity across a wider spectrum of SC types compared to either agent alone, as evidenced by Zhu et al. [118] in studies conducted on senescent human preadipocytes, HUVECs, primary mouse embryonic fibroblasts (MEFs), and bone-marrow-derived murine mesenchymal stem cells. D + Q in vivo reduced the number of SCs in naturally aged, radiation-exposed, and progeroid Ercc1^−/Δ^ mice, improving cardiovascular function and exercise capacity. Moreover, intermittent dosing in Ercc1^−/Δ^ mice extended healthspan, delaying the onset of age-related phenotypes and pathologies, such as osteoporosis and intervertebral disc degeneration.

D + Q treatment alleviated LPS-induced senescence in HUVECs, inhibiting SASP via the TRAF6-MAPK-NF-κB pathway, by upregulating m6A reader YTHDF2 that is a regulator of the stability of MAP2K4 and MAP4K4 mRNAs [119].

Kaempferol (3,4′,5,7-tetrahydroxyflavone) is a natural flavonol, found in a variety of plants and plant-derived foods including, beans, capers, saffron, kale, spinach, ginger, and broccoli.

Kaempferol treatment improved IL-1β-induced senescent nucleus pulposus cells, inhibiting cell senescence and apoptosis, increasing ECM production, and decreasing ECM degradation partially restoring the alterations in ECM production and degradation [120].

Aged porcine oocytes treated with KAE have a blastocyst production rate significantly higher respect to untreated aging oocytes, have a higher expression of the embryonic pluripotency-related Oct4, NANOG, and ITGA5 genes, and maintain the mitochondrial membrane potential [121].

In bleomycin-induced senescent BJ cells (human foreskin fibroblast), treatment with Kaempferol reduced the production of SASP components (IL-6, IL-8, IL-1β), without affecting significantly senescence itself. This inhibition seems to be mediated by interfering with the IRAK1/IκBα/NF-κB p65 pathway, since Kaempferol significantly reduced IκBζ mRNA and protein levels [92].

KAE tetrasaccharides are a KAE derivative and natural inhibitors of pyruvate dehydrogenase kinase 1 (PDK1) that can prevent induced senescence in normal human dermal fibroblasts, as indicated by the reduction of SASP components and proinflammatory cytokines, and by the decrease of p16, p21, p65, and IkB phosphorylation [122].

Similarly to fisetin, Kaempferol reduced the intracellular ROS accumulation and increased the lifespan of *C. elegans*, attenuating the accumulation of lipofuscin, an age-dependent marker [123].

Rutin, a flavonoid glycoside also known as quercetin-3-O-rutinoside or vitamin P, plays a significant role in modulating cellular senescence through its multifaceted biological activities. Its antisenescent properties are largely attributed to its strong antioxidant and anti-inflammatory effects, as well as its enzymatic conversion into quercetin, a more potent aglycone with superior pharmacological efficacy [124].

As observed in a cellular model using a primary normal human prostate stromal cell line (PSC27), treated with bleomycin to induce senescence, Rutin has been shown to attenuate the SASP by inhibiting the acute stress-associated phenotype (ASAP), an early senescence response that precedes full SASP expression. Specifically, rutin interferes with the activation of ataxia-telangiectasia mutated (ATM) kinase and its downstream interactions with hypoxia-inducible factor 1-alpha (HIF-1α) and TNF receptor-associated factor 6 (TRAF6)—key regulators in the development of both ASAP and SASP. The modulation of senescence by rutin has notable implications in the tumor microenvironment. In prostate cancer models, conditioned media from senescent stromal cells enhanced malignant behaviors such as proliferation, invasion, migration, and chemoresistance. Remarkably, rutin reversed these effects by downregulating SASP-associated signaling [125]. Rutin also demonstrated protective effects in age-related vascular and neurodegenerative conditions. Diabetic mice exhibit significantly increased atherosclerotic plaque burden in the aortic arteries, accompanied by a reduction in vascular smooth muscle cells (VSMCs) and an elevated proportion of senescent cells within the plaque, compared to mice on control diets. Treatment with rutin markedly improved glucose and lipid metabolic disturbances associated with diabetes and additionally contributed to the attenuation of atherosclerotic plaque formation, reducing atherosclerotic burden and SCs accumulation while enhancing the VSMC content in plaques at the aortic root. In vitro studies further demonstrated that rutin mitigated H_2_O_2_-induced premature senescence in VSMCs, potentially through mechanisms involving the inhibition of oxidative stress and preservation of telomere integrity [126].

In vivo experiments demonstrated that rutin is able to increase the lifespan of *C. elegans* and its egg deposition. In D-Gal-induced aging mice, after rutin treatment, an improvement could be observed in the exercise capacity and a reduction in brain tissue ROS and malondialdehyde [127].

Fisetin (3,7,3′,4′-tetrahydroxyflavone) is a dietary flavonol found at low concentrations in fruits and vegetables such as cucumber, apple, grape, and onion, with the highest concentration being found in strawberries [128].

Zhu et al. were the first to demonstrate the senolytic effect of fisetin. They demonstrated that fisetin affects the viability of senescent HUVECs without having effect on proliferating cells [15].

Fisetin decreased cellular senescence and SASP factors in human endothelial cell cultures. Oral intermittent supplementation of fisetin to old mice improved arterial function mediated by increasing NO bioavailability and reducing total and mitochondrial ROS [129].

In both progeroid (Ercc1^−/∆^) and aged wild-type mice, acute or intermittent fisetin treatment significantly reduced senescence markers across multiple tissues. This reduction was also observed in specific cell types within murine and human adipose tissue, highlighting its cell-type specificity.

In vivo, dietary fisetin supplementation in progeroid and aged wild-type mice led to decreased p16 expression and reduced levels of SASP factors in adipose tissue. In vitro, treatment with 5 mM fisetin for 48 h significantly reduced SA-β-Gal positivity in murine embryonic fibroblasts driven into senescence by oxidative stress. Similarly, fisetin treatment effectively diminished senescence markers in human fibroblasts exposed to the genotoxic agent etoposide. Importantly, chronic fisetin administration in wild-type natural aged mice extended median and maximum lifespan, restored tissue homeostasis, and alleviated age-related pathologies [130]. Fisetin treatment significantly decreased SA-β-Gal+ and GL13+ (lipofuscin) senescent neurons, astrocytes, and microglia in both grey and white matter of the cerebral brain cortex and non-Cornu Ammonis area of the hippocampus of old sheep. Fisetin treatment also significantly decreased plasma S100B, a neuronal aging and injury marker. In addition, fisetin treatment reduced significantly the expression of senescence-associated genes (GLB1) in liver, and inflammasome components (NLRP3 and TREM2) in other organs, including the lung and liver [131].

Human primary adipose-derived stem cells (ADSCs) are a population of fat-resident mesenchymal stem cells widely used in regenerative medicine. During the culture expansion, these cells progressively acquire hallmark features of cellular senescence, including increased SA-β-Gal activity, elevated ROS and the presence of senescence-associated heterochromatin foci (SAHF). Fisetin reduced these senescence markers in a dose-dependent manner while preserving the differentiation capacity of the expanded ADSCs [132].

### 3.4. Tannins

Tannins are high-molecular-weight polyphenolic compounds broadly classified into two main categories: hydrolysable tannins and condensed tannins (also known as proanthocyanidins). They are abundantly present in various plant-derived foods and beverages, including tea, red wine, berries, nuts, legumes, and certain tree barks. Hydrolysable tannins are esters of gallic acid (gallotannins) or ellagic acid (ellagitannins) with a polyol core, typically glucose. In contrast, condensed tannins are oligomers or polymers of flavan-3-ol units (e.g., catechin, epicatechin), linked predominantly via C4 → C8 or C4 → C6 interflavan bonds, rendering them non-hydrolysable under mild conditions.

Procyanidin C1 (PCC1), a trimeric B-type epicatechin oligomer isolated from grape seed extract, has been identified as a potent phytochemical senotherapeutic agent with enhanced selectivity and efficacy against a broad spectrum of SCs phenotypes and senescence-inducing stimuli, surpassing the activity profiles of several canonical senolytics. PCC1 exerts a concentration-dependent biphasic effect: at sub-cytotoxic concentrations, it functions as a senomorphic compound by attenuating the SASP, whereas at elevated concentrations, it induces apoptosis selectively in SCs, conferring senolytic activity. Mechanistically, the senolytic action of PCC1 is attributed to its capacity to modulate mitochondrial membrane potential and activate intrinsic apoptotic pathways, thereby facilitating SCs clearance. Preclinical studies in mice have demonstrated that PCC1 treatment leads to reduced SCs burden in various tissue of aged mice, extended healthspan, and amelioration of age-associated physiological decline in motor coordination, balance, endurance capacity, muscle strength, and spontaneous exploratory behavior in aged murine models [133].

### 3.5. Other Polyphenols

Gingerenone A is a diarylheptanoid polyphenol present in *Zingiber officinale*. Treatment with 20 μM gingerenone A elicited a significant reduction in the viability of senescent WI-38 human diploid fibroblasts, while exhibiting minimal cytotoxicity toward proliferating counterparts. This senolytic effect was accompanied by a marked attenuation in the secretion of key SASP components, including IL-6, C-C motif chemokine ligand 2 (CCL2/MCP-1), and interferon-γ-induced protein 10 (IP-10). Concurrently, gingerenone A treatment led to an upregulation of the anti-inflammatory cytokines IL-10 and IL-13, indicating a dual action of SASP suppression and promotion of a more anti-inflammatory secretory profile.

Mechanistically, the senolytic activity of gingerenone A appears to be mediated through the downregulation of Bcl-xL, a key antiapoptotic member of the Bcl-2 protein family, leading to enhanced activation of caspase-3 and subsequent apoptotic clearance of senescent cells [134].

The Mediterranean diet has been extensively correlated with increased longevity and reduced incidence of age-related pathologies. A principal bioactive component of this diet is extra virgin olive oil (EVOO), which is rich in phenolic compounds such as hydroxytyrosol (HT; 2-(3,4-dihydroxyphenyl) ethanol) and oleuropein (OLE). These polyphenols exhibit potent antioxidant, anti-inflammatory, and neuroprotective activities. HT and its derivatives represent the predominant hydrophilic phenolic fraction in EVOO and have been shown to modulate multiple hallmarks of aging. In UVA-irradiated HDFs, HT reduced SA-β-gal activity in a dose-dependent fashion, and downregulated matrix metalloproteinases MMP-1 and MMP-3. Furthermore, HT significantly decreased the mRNA expression of inflammatory cytokines IL-1β, IL-6, and IL-8, as confirmed by quantitative RT-PCR analysis [135]. Similarly, both HT and OLE have been demonstrated in vitro to mitigate senescence phenotypes, as evidenced by reduced SA-β-gal staining, lower p16 expression, and diminished SASP factor levels in presenescent MRC5 human lung fibroblasts and neonatal HDFs [136]. OLE has also been reported to enhance proteasome activity, thereby delaying the onset of cellular senescence in human fibroblasts. Chronic OLE exposure in early-passage human embryonic fibroblasts attenuated ROS accumulation, inhibited senescence-associated morphological alterations, and decreased cell mortality rates. Moreover, HT has been implicated in the activation of pro-longevity molecular signaling cascades, including AMPK and SIRT1 pathways, as well as in the promotion of mitochondrial biogenesis, enhancement of autophagic flux, stimulation of DNA repair mechanisms, ROS detoxification, and epigenetic regulation [137].

**Figure 4:**
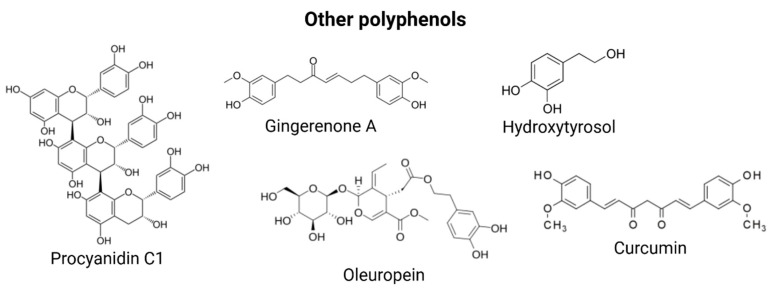
Structural formulas of other polyphenols described.

Curcumin, a natural polyphenol derived from the rhizome of *Curcuma longa* (commonly known as turmeric), has garnered significant attention for its potential role in promoting healthy aging. Curcumin has been shown to extend the lifespan of two distinct strains of *Drosophila melanogaster*, with these longevity benefits accompanied by enhanced resistance to oxidative stress, improved locomotor function, and notable chemopreventive effects. Interestingly, the extent of lifespan extension was found to be both genotype- and gender-specific, suggesting that curcumin’s effects are influenced by underlying genetic and biological differences. Curcumin also modulated the expression of several key genes associated with aging and stress response in *D. melanogaster* (mth, thor, JNK, InR) [138]. Curcumin reduced intracellular ROS and lipofuscin, enhanced resistance to oxidative and thermal stress, and extended the lifespan of wild-type *C. elegans* [139]. At the molecular level, curcumin’s antiaging effects are closely associated with several key signaling pathways involved in cellular stress responses and longevity regulation. In animal studies, particularly in mice undergoing endurance training, curcumin supplementation has been shown to enhance AMPK phosphorylation in skeletal muscle, increase SIRT1 expression, and stimulate mitochondrial biogenesis phosphorylation CREB and LKB-1-factors crucial for maintaining metabolic health and delaying age-associated decline [140]. Moreover, curcumin modulates inflammatory responses by activating sirtuins and suppressing the NF-κB pathway. It achieves this in part by inhibiting the acetylation of the NF-κB subunit p65, thereby reducing the transcription of pro-inflammatory cytokines [141]. Additionally, through activation of the Nrf2-Keap1 pathway, curcumin enhances cellular antioxidant defenses and reduces oxidative stress, which plays a central role in the pathophysiology of numerous chronic diseases. It can also downregulate TNF-α, further mitigating systemic inflammation [142]. In the context of neurodegenerative diseases such as AD and PD, curcumin has demonstrated a broad spectrum of beneficial effects. Epidemiological studies have also drawn connections between curcumin supplementation and improved cognitive performance, suggesting its neuroprotective potential. These include antioxidative, antiapoptotic, and anti-inflammatory actions, as well as the ability to promote neurogenesis, which collectively may slow disease progression and improve neurological outcomes [143]. In a study by Cox et al. [144], both acute and chronic curcumin interventions in 60 healthy adults aged 60–85 resulted in significant improvements in learning and memory, underscoring its therapeutic potential for cognitive aging. One hour after administration, curcumin significantly improved performance on sustained attention and working memory tasks, compared with placebo. Structural formulas of these polyphenols are illustrated in Figure 4.

## 4. Discussion

While advancements in medical science have significantly extended human lifespan, they have also led to a parallel increase in the prevalence of chronic, age-associated diseases. One of the principal contributors to the aging process is the accumulation of SCs—cells that have permanently exited the cell cycle, exhibit resistance to apoptosis, and yet remain metabolically active. Although transient senescence plays a critical physiological role in processes such as wound healing and tissue remodeling, chronic accumulation of SCs, as seen in aging, results in the sustained secretion of pro-inflammatory cytokines, chemokines, growth factors, and matrix-remodeling enzymes collectively known as the SASP. This pro-inflammatory microenvironment disrupts tissue homeostasis, contributes to organ dysfunction, and facilitates the pathogenesis of age-related diseases, including cancer. Therapeutics strategies targeting SCs have gained momentum, with senolytics and senomorphics showing significant promise in preclinical models. Senolytics selectively induce apoptosis in SCs, thereby reducing their burden and mitigating associated tissue dysfunction. Senomorphics, in contrast, suppress the deleterious effects of SCs without inducing cell death, primarily through modulation of the SASP. Notably, several naturally occurring polyphenolic flavonoids—such as quercetin, fisetin, and curcumin (senolytics) and resveratrol, kaempferol, apigenin, and EGCG (senomorphics)—exhibit potent antioxidant and anti-inflammatory properties, rendering them attractive candidates for senotherapeutic interventions. Intriguingly, these compounds have been shown not only to alleviate cellular senescence in vitro and in vivo but also to extend the lifespan of various model organisms. Despite these promising findings, the translation of flavonoid-based senotherapeutics into clinical practice faces several challenges. Chief among them is the inherently low bioavailability of polyphenolic flavonoids, which is attributed to their poor aqueous solubility, limited intestinal permeability, and extensive first-pass metabolism into inactive glucuronide and sulphate conjugates. Furthermore, interindividual variability in genetic polymorphisms affecting flavonoid metabolism and transport may lead to inconsistent therapeutic outcomes. As such, innovative strategies are urgently needed to overcome these pharmacokinetic limitations. Potential solutions include co-administration with bioavailability enhancers, structural modification of parent compounds, and the application of advanced drug delivery systems such as nanoparticle encapsulation, liposomal carriers, cocrystallization, and other nanotechnological platforms. Large-scale, randomized clinical trials are also essential to validate the efficacy, safety, and mechanistic action of these compounds in human populations and to pave the way for the development of next-generation senotherapeutics. Polyphenols are generally considered safe for healthy individuals when consumed as part of balanced and varied diet. As such, incorporating polyphenol-rich foods—particularly unprocessed or minimal processed items such as fruits, vegetables, nuts, herbs, spices, teas, and natural juices—into daily diet is highly recommended. However, attention must be paid to the consumption of supplements or concentrated plant extracts. These products contain unnaturally large quantities of polyphenols, frequently in the form of purified aglycones rather than the glycosylated forms typically found in whole foods—levels that may pose health risks [145]. The optimal intake of polyphenols is not yet definitively established due to variations in individual metabolism, dietary sources, and bioavailability. General recommendations advise an average daily intake of 1 g/day approximately, mainly from fruit, vegetables, tea, coffee, and wine. Indeed, healthy benefits have been observed with intakes ranging from 500 to 1500 mg/day, this goal can be achieved with daily consumption of at least 5 portions of vegetables/fruits [146]. Table 1 summarizes the average content of antiageing polyphenols in the foods in which they are most abundant.

## 5. Conclusions

Targeting SCs represents a promising strategy to counteract age-related diseases. Naturally occurring flavonoids, with senolytic and senomorphic properties, have demonstrated efficacy in preclinical models by modulating the SASP and promoting tissue homeostasis. Future research should focus on improving delivery systems and conducting robust clinical trials to validate their therapeutic potential in humans and support the development of effective senotherapeutics.

## Figures and Tables

**Table 1:** Mean content of antiageing polyphenols in foods.

Polyphenol	Source	Mean Content	References
(−)-Epigallocatechin 3-O-Gallate	Tea [Black], infusion	9.12 mg/100 mL	[147,148]
	Tea [Green], infusion	27.16 mg/100 mL	[147,149]
	Tea [Oolong], infusion	17.89 mg/100 mL	[150,151]
Procyanidin Trimer C1	Chocolate, dark	26.00 mg/100 g FW	[152]
	Apple [Dessert], pure juice	29.97 mg/100 mL	[153,154]
	Plum, fresh	10.01 mg/100 g FW	[155]
Hesperidin	Lemon, pure juice	17.81 mg/100 mL	[156,157]
	Orange [Blond], pure juice	25.85 mg/100 mL	[158,159]
	Orange [Blood], pure juice	43.61 mg/100 mL	[160]
	Peppermint, dried	480.65 mg/100 g FW	[161]
Naringenin	Mexican oregano, dried	372.00 mg/100 g FW	[162]
Apigenin	Italian oregano, fresh	3.50 mg/100 g FW	[163]
	Marjoram, dried	4.40 mg/100 g FW	[164]
	Common sage, fresh	2.40 mg/100 g FW	[163]
Luteolin	Common sage, fresh	33.40 mg/100 g FW	[163]
	Common thyme, fresh	39.50 mg/100 g FW	[163]
	Mexican oregano, dried	56.33 mg/100 g FW	[162]
	Globe artichoke, heads, raw	42.10 mg/100 g FW	[165]
Nobiletin	Orange [Blood], pure juice	0.31 mg/100 mL	[166]
Tangeretin	Orange [Blood], pure juice	0.04 mg/100 mL	[166]
Kaempferol	Capers	104.29 mg/100 g FW	[167]
	Cloves	23.80 mg/100 g FW	[168]
	Cumin	38.60 mg/100 g FW	[168]
Quercetin	Chocolate, dark	25.00 mg/100 g FW	[169]
	Black elderberry	42.00 mg/100 g FW	[170]
	Mexican oregano, dried	42.00 mg/100 g FW	[162]
	Capers	32.82 mg/100 g FW	[167]
	Cloves	28.40 mg/100 g FW	[168]
Genistein	Soy paste, miso	7.25 mg/100 g FW	[171,172,173]
	Soy, tempe	10.00 mg/100 g FW	[174,175,176]
	Soy, tofu, fermented	9.68 mg/100 g FW	[171,173]
Curcumin	Turmeric, dried	2213.57 mg/100 g FW	[177,178]
	Curry, powder	285.26 mg/100 g FW	[177]
Hydroxytyrosol	Olive [Black], raw	65.93 mg/100 g FW	[179,180,181]
	Olive [Green], raw	55.57 mg/100 g FW	[179,182]
Oleuropein-Aglycone	Olive, oil, refined	12.54 mg/100 g FW	[183]
	Olive [Black], raw	81.82 mg/100 g FW	[179,184]
	Olive [Green], raw	58.50 mg/100 g FW	[179]
	Olive, oil, virgin	12.06 mg/100 g FW	[183,185]
Gallic Acid	Walnut, liquor	15.15 mg/100 mL	[186]
	Wine [Red]	3.59 mg/100 mL	[187,188,189]
	Cloves	458.19 mg/100 g FW	[190]
	Chestnut, raw	479.78 mg/100 g FW	[191]
	Chicory [Green], raw	25.84 mg/100 g FW	[192]
Protocatechuic Acid	Sorghum, whole grain	2.55 mg/100 g FW	[193]
	Date, dried	4.94 mg/100 g FW	[194]
	Star anise	32.20 mg/100 g FW	[168]
	Chicory [Red], raw	16.78 mg/100 g FW	[192]
Caffeic Acid	Common sage, dried	26.40 mg/100 g FW	[195]
	Spearmint, dried	25.00 mg/100 g FW	[195]
	Ceylan cinnamon	24.20 mg/100 g FW	[168]
	Star anise	20.20 mg/100 g FW	[168]
Resveratrol	Muscadine grape, red wine	3.02 mg/100 mL	[196]
	European cranberry	1.92 mg/100 g FW	[197]
	Lingonberry, raw	3.00 mg/100 g FW	[197]
	Wine [Red]	0.27 mg/100 mL	[188,189,198]

## Data Availability

Data are contained within this article.

## Supplementary Materials

The following supporting information can be downloaded at: https://www.mdpi.com/article/10.3390/nu17101716/s1, Table S1: Summary table of studies on the senotherapeutic effects of polyphenols discussed in the article.

## Abbreviations

The following abbreviations are used in this manuscript:

AD	Alzheimer’s disease
ADSCs	adipose-derived stem cells
AMPK	AMP-activated protein kinase
ASAP	acute stress-associated phenotype
ATM	ataxia-telangiectasia mutated
Bax	Bcl-2 associated X protein
Bcl	B Cell Lymphoma
BMSCs	bone-marrow-derived mesenchymal stem cells
CCL2	C-C motif chemokine ligand 2
COX-2	Cyclooxygenase-2
DNA-SCARS	DNA segments with chromatin alterations reinforcing senescence
ECs	endothelial cells
EGCG	Epigallocatechin gallate
ERRα	estrogen-related receptor alpha
EVOO	extra virgin olive oil
EVs	extracellular vesicles
GA	gallic acid
GSE	grape seed extract
HaCaT	human keratinocytes
HDF	human dermal fibroblasts
HEI-OC1	House Ear Institute-Organ of Corti 1 cells
hHBMCs	human bone-marrow-derived mesenchymal stem cells
HIF-1α	hypoxia-inducible factor 1-alpha
HIIT	high-intensity interval training
hMSCs	human mesenchymal stem cells
htNSCs	hypothalamic neural stem cells
hUC-MSC	human umbilical cord mesenchymal stem cell
HUVECs	human umbilical vein endothelial cells
IFN-γ	Interferon Gamma
IP-10	interferon-γ-induced protein 10
JNK	c-Jun N-terminal kinase
KAE	Kaempferol (3,4′,5,7-tetrahydroxyflavone)
Ki-67	Kiel 67
LKB1	liver kinase B1
lncRNAs	long non-coding RNAs
LPS	lipopolysaccharide
MMP	Matrix Metalloproteinase
MSCs	mesenchymal stromal cells
mTOR	mammalian Target Of Rapamycin
NF-κB	nuclear factor kappa-light-chain-enhancer of activated B cells
NO	Nitric Oxide
ROS	Reactive Oxygen Species
Nrf2	nuclear factor erythroid 2–related factor 2
OA	osteoarthritis
OLE	oleuropein
OVX-BMMSCs	Bone marrow mesenchymal stem cells from ovariectomized rats
ox-LDL	oxidized low-density lipoprotein
p38 MAPK	p38 mitogen-activated protein kinases
PCA	protocatechuic acid
PCC1	Procyanidin C1
PD	Parkinson’s disease
PDK1	pyruvate dehydrogenase kinase 1
PGC-1α	proliferator-activated receptor gamma coactivator 1-alpha
PIC	Piceatannol
PM2.5	Particular matter 2.5
RES	resveratrol
SAHF	senescence-associated heterochromatin foci
SAMP8	senescence-accelerated mouse prone 8
SASP	senescence-associated secretory phenotype
SA-β-gal	senescence-associated beta-galactosidase
SCs	senescent cells
SDF	senescence-associated DNA damage foci
SIRT	sirtuin
TF2A	theaflavin-3-gallate
TGF-β	Transforming Growth Factor beta
TNF-α	Tumor Necrosis Factor Alpha
TRAF6	TNF receptor-associated factor 6
UV	ultraviolet
VSMCs	vascular smooth muscle cells
WS	Werner syndrome
