# Supplementary material for: The Antiaging Potential of Dietary Plant-Based Polyphenols: A Review on Their Role in Cellular Senescence Modulation
Source: Nutrients. 2025 May 19;17(10):1716. doi: 10.3390/nu17101716 (PMC12114605; doi:10.3390/nu17101716)
Supplement: Supplementary file 1 [file nutrients-17-01716-s001.zip › nutrients-3618402-supplementary.pdf]

Class	Compound	Models	Experimental methods	Effects	References
**Phenolic acids**	Gallic Acid (GA)	**A)** Rat embryonic fibroblast (REF) cells<br>**B)** Human mesenchymal stem cells (hMSCs)	**A)** Induction to senescence with 600 μM of H_2O_2 and treatment with 554.25 μM of GA.<br>**B)** Induction of Werner syndrome in hMSCs and treatment with GA.	**A)** Reduction of β-gal activity, inflammatory cytokines and oxidative stress markers.<br>**B)** Delaying cellular replicative senescence, reduction of senescence markers, ROS and cellular apoptosis and extended telomere length.	**A)** [26]<br>**B)** [27]
	Protochatechuic Acid (PCA)	**A)** *Caenorhabditis elegans*<br>**B)** Human dermal fibroblasts (HDFs)	**A)** Treatment of *C. elegans* with 100 or 200 μM of PCA.<br>**B)** LPS (1 μg/mL)-induced HDFs were treated with PCA (0–100 μM).	**A)** Increasing in lifespan and enhanced resistance to different stress.<br>**B)** Reduction of intracellular ROS and number of β-gal-positive cells, and regulation of ECM-related expression.	**A)** [28]<br>**B)** [29]
	Vanillic Acid (VA)	**A)** *Caenorhabditis elegans*	**A)** Treatment of *C. elegans* with 0.5, 1 e 5 mM of VA.	**A)** Increasing thermotolerance, reduction of protein aggregation and extending lifespan.	**A)** [30]
	Caffeic Acid (CA)	**A)** Male Sprague–Dawley rats<br>**B)** Human dermal fibroblasts (HDFs)	**A)** Rats were treated with D-galactose (50 mg/kg) then treated with CA (20 or 40 mg/kg).<br>**B)** HDFs (young and aged) were treated with CA (6.25-12.5 μg/mL) alone or in co-culture with human keratinocytes (HaCaT).	**A)** Attenuation of overexpression of apoptotic proteins Bcl-2, Bax and caspase-3, improving brain aging.<br>**B)** Reduction of β-gal activity, induction of collagen synthesis, inhibition of IL-6, MMP-1, MMP-9, IL-8 and in co-culture protecting against photoaging caused by UV rays.	**A)** [31]<br>**B)** [32]
**Stilbenes**	Resveratrol (RES)	**A)** 18-month-old and aged male C57BL/6 mice<br>**B)** Leukocytes isolated from human donors (20–39 years, 40–59 years, and 60–80 years)<br>**C)** 20-months-old male Wistar rats<br>**D)** 4 to 8 week male C57Bl/6J mice.<br>**E)** Nothobranchius guentheri<br>**F)** 4 months old and 24 months old male Wistar-albino rats<br>**G)** Male C57BL/6NIA mice<br>**H)** Transgenic AD mice and human umbilical cord-derived mesenchymal stem cells (hUC-MSCs)<br>**I)** Human dermal papilla cells (DP)<br>**J)** hUC-MSC cells<br>**K)** Healthy older adults (50–80 years) patients	**A)** Mice were fed with a mixture of resveratrol (40 mg/kg) and normal chow for 6 months.<br>**B)** Leukocytes were treated with RES (5 μM) for 24 h and were exposed to H_2O_2 (150 μM) for 24 h.<br>**C)** Rats received RES (10 mg/kg/day) via oral gavage for 6 weeks alone or in combination with swimming high-intensity interval training.<br>**D)** Mice were fed with RES (4g/kg of food) mixed with either powdered chow or high fat diet.<br>**E)** Fish were fed with RES-supplemented food (200 μg/g food).<br>**F)** RES (50 mg/kg/day) was given to mice orally for 12 weeks.<br>**G)** Mice were fed with a standard or modified to provide 60% of calories from fat (HC) plus 0.01% or 0.04% RES.<br>**H)** hUC-MSCs were transplanted in AD mice model, that received RES by oral gavage (200 mg/kg/day) for 8 weeks.<br>**I)** PM2.5 (50 μg/ml)-senescence induced DP were treated with RES (50 μM or 100 μM)<br>**J)** hUC-MSC were subjected 0.1, 1 and 2.5 μM RES for 6 days.<br>**K)** Subjects follow a daily intake of four	**A)** Increasing of Nrf2-HO-1-NOQ-1 and SIRT1-AMPK-PGC-1α signaling, improving oxidative stress and mitochondrial dysfunction.<br>**B)** Reduced the levels of TNF-α and IL-6, by activating Nrf2 pathway.<br>**C)** In combination with exercise enhance the expression of Sirt 4, Sirt 5, SOD 1 and SOD 2.<br>**D)** Protection from obesity, increasing mitochondrial function, reduction of inflammation through SIRT1 activity.<br>**E)** Inhibiting SASP through SIRT1/NF-κB signaling pathway and delayed aging.<br>**F)** Reduction of TNF-α, improving chronic neuroinflammation, spatial learning and memory<br>**G)** Reduction of albuminuria, inflammation and apoptosis in the vascular endothelium, cataract formation, increased aortic elasticity, motor coordination and preserved bone mineral density.<br>**H)** Enhanced neurogenesis and	**A)** [35]<br>**B)** [37]<br>**C)** [38]<br>**D)** [39]<br>**E)** [40]<br>**F)** [45]<br>**G)** [47]<br>**H)** [48]<br>**I)** [49]<br>**J)** [51]<br>**K)** [52]

		capsules (in total 200 mg of resveratrol and 320 mg of quercetin) over a period of 26 weeks.	improved learning and memory functions through sirt1 activity.<br>**I)** Reduction of SA-β-gal expression, mRNA levels of SASP proteins IL1α, IL7, IL8 and CXCL1 and p21, p16 levels.<br>**J)** Enhancing cell viability and proliferation, through upregulation of SIRT1 e PCNA and downregulating of p53 and p16.<br>**K)** Improved glucose metabolism, hippocampal connectivity, and memory performance.	
Piceatannol (PIC)	**A)** Human mesenchymal stem cells (hMSCs)<br>**B)** Human fetal lung fibroblast (TIG-3-20) cells<br>**C)** Male 8-week-old and 48-week-old C57BL/6 mice<br>**D)** *Caenorhabditis elegans*<br>**E)** Male Kunming mice and 21-month-old male C57BL/6J mice	**A)** Induction to acute senescence with 300 μM of H_2O_2 for 30 min or to chronic senescence with 30 day of in vitro cultivation and treatment with PIC (0.001 μM-10 μM).<br>**B)** TIG-3-20 were irradiated with X ray at a dose of 1.51 and 2.64 Gy/min and then treated with PIC (0, 0.125, 1.25, and 12.5 μM).<br>**C)** 48-week-old geriatric mice group were treated with PIC (2.5, 5, 10 mg/kg).<br>**D)** Different strains of *C.elegans* were treated with PIC (50 e 100 μM).<br>**E)** Aging in Kunning mice was induced by administering D-gal (150 mg/kg) daily for eight days + PIC (20 mg/kg) for 8 weeks. Aged C57BL/6J mice were orally administered with piceatannol (20 mg/kg) for 4 weeks.	**A)** Impaired acute and replicative senescence reducing respectively the expression of P53–P21, RB1 pathways, and RB2–P16–P21–P27 pathways. Downregulation of SIRT1.<br>**B)** Pre-treatment with PIC suppress radiation-induced DSB reducing ROS. Post-irradiation PIC treatment enhance efficiency of ATM-mediated DSB repair and reduces SA-β-gal levels.<br>**C)** Improved age-related hearing loss by modulating the inflammatory response and reduced pyroptosis through caspase-11-GSDMD pathway.<br>**D)** Extend the lifespan and enhance stress resistance through DAF-16 and sir-2.1.<br>**E)** Maintain spontaneous motor activity and improve learning and memory ability through activation of the Nrf2 pathway.	**A)** [55]<br>**B)** [56]<br>**C)** [57]<br>**D)** [59]<br>**E)** [60]

**Flavanols**	Epigallocatechin gallate (EGCG)	**A)** 3T3-L1 preadipocytes cells<br>**B)** 3T3-L1 preadipocytes cells<br>**C)** Young (4 months old) and aged (18 months old) male C57BL/6J mice and 3T3-L1 preadipocytes cells.<br>**D)** Human umbilical vein endothelial cells (HUVECs) and THP1 monocytes<br>**E)** Rat vascular smooth muscle cells (RVSMCs), human dermal fibroblasts (HDFs), and human articular chondrocytes (HACs)<br>**F)** Human mesenchymal stem cells (hMSCs)	**A)** 3T3-L1 were exposed to BrdU (100 μM) for 8 days and then to EGCG (50-10 μM) or EGCG (40%)-resveratrol (40%)-spermidine (20%) mix (20-30 μM).<br>**B)** 3T3-L1 were treated with H_2O_2 (150 μM) in combination to EGCG (50-100 μM).<br>**C)** 3T3-L1 were exposed to H_2O_2 (150 μM). Macrophage cells from the mice were pretreated with EGCG (10 μM) for 24 h and then treated with culture medium from young and senescent preadipocytes.<br>**D)** ECs were treated with etoposide (10 μM) for 24 h, subsequently, were treated with EGCG (100 μM) for 24 h. ECs treated and control were co-cultured with THP1 for 24 h, then THP1 were stimulated with LPS.<br>**E)** Primary cells were serially passaged. From passage 3 to 20, EGCG (50 μM) was added to the culture media of RVSMCs and HACs with media refreshment during serial culture. HDFs were subcultured serially from passage 7 to ≥40 in the presence of EGCG (50/100 μM).<br>**F)** hMSCs were pre-incubated with EGCG (50 and 100 μM) for 6 h and then exposed to 200 μM H_2O_2 for 2 h.	**A)** Inhibition of ROS, NF-kB, IL-6 and CDKN1a through SIRT3.<br>**B)** Reduction of PI3K/Akt/mTOR signaling and ROS, iNOS, COX-2, NF-κB, SASP and Bcl-2 levels.<br>**C)** Modulation of activation and inflammatory state of old macrophage cells and reduction of p53, p21 and p16.<br>**D)** Reduction of SA-β-gal and senescence markers. Modulation of comunication between senescent ECs and monocytes, attenuating pro-inflammatory responses.<br>**E)** Prevent replicative senescence, suppressing the p53 acetylation.<br>**F)** Reduction of oxidative stress, suppressing p53/p21 pathway and upregulating Nrf2 expression.	**A)** [65]<br>**B)** [66]<br>**C)** [67]<br>**D)** [68]<br>**E)** [69]<br>**F)** [70]
	Theaflavin 3-gallate (TF2A)	**A)** Primary hypothalamic neural stem cells (htNSCs) from the hypothalami of 3- and 18-month-old mice, C57/BL6J and Gm31629 knockout mice<br>**B)** Gm31629 knockout mice, 3-month-old and 12-month-old C57BL/6J male mice and bone marrow mesenchymal stem cells (BMSCs) from these mice	**A)** Cells were treated with buthionine sulfoximine (100 μM) for 48 h then with T2FA for 72 h. TF2A (8 mg/kg/day) was administered by oral gavage for six months to mice.<br>**B)** Mice were treated with TF2A (8 mg/kg/day) by gavage for 3 weeks before the establishment of bone regeneration model, with surgical procedure. After that, TF2A treatment continued for one week. Third-passage BMSCs were treated with TF2A.	**A)** Mimic and modulate the gene expression of lncRNAs improving aging-associated pathology and bone regeneration, stabilizing YB-1.<br>**B)** *In vitro* reduced the expression of p16 and mimicked the ability of Gm31629 to increase YB-1, enhancing osteogenic differentiation. *In vivo* increased bone regeneration in aging populations.	**A)** [71]<br>**B)** [72]
**Isoflavones**	Genistein	**A)** Vascular smooth muscle cell (VSMC) from Sprague–Dawley rats<br>**B)** 20-week-old female Sprague-Dawley rats and bone marrow mesenchymal stem cells from ovariectomized (OVX-BMMSCs) and control (Sham-BMMSCs) rats<br>**C)** Human umbilical vein endothelial cells (HUVECs)	**A)** VSMC were stimulated with adriamycin (500 nM) for 4 h, then treated with genistein (5, 10 and 20 μM).<br>**B)** Rats were received operation to establish the OVX-OP model. 8 weeks after surgery, rats received oral genistein (50 mg/kg body weight) treatment once daily. OVX-BMMSCs were treated with genistein (1 μM or 10^{-2} μM) for 3 days.<br>**C)** HUVECs were pretreated with genistein (1000 nM) for 30 min. After that, cells were exposed to oxidized low-density lipoprotein (ox-LDL) (50 mg/L) for another 12 h. Furthermore, HUVECs were pretreated with BafA1 (50 nM) for 30 min, and subsequently incubated with genistein and then ox-LDL.	**A)** Inhibition of mTOR and activation of autophagy via LKB1–AMPK.<br>**B)** Reduction of p53, p21, p16, intracellular ROS levels and restoration of mitochondrial homeostasis in an ERR*α*-dependent manner. Reduction trabecular bone loss, downregulation of p16, and upregulation of SIRT3 and PGC-1*α* *in vivo*.<br>**C)** Decreased p16 and p21 protein levels and SA-β-gal activity, and increased LC3-II and decreased tP62 protein levels through the SIRT1/LKB1/AMPK pathway.	**A)** [74]<br>**B)** [75]<br>**C)** [76]

**Flavanones**	Hesperidin	**A)** MRC-5 human lung fibroblasts<br>**B)** Human articular chondrocytes<br>**C)** 20-month-old male Wistar rats	**A)** MRC-5 cells were induced to senescence with doxorubicin (100 nM) and then treated with hesperidin (10 μg/ml, 25 μg/ml, 50 μg/ml).<br>**B)** Chondrocytes were harvested from patients with knee osteoarthritis, then were treated with 0.5 mM H_2O_2 for 30 min and subsequently were cultured in medium containing hesperidin (5 or 10 μM).<br>**C)** Rats were fed with casein-based diet containing 0.4% calcium and 0.3% phosphorus, supplemented with 0.5% hesperidin, 0.5% naringin or a mix of both flavanones (0.25% each) for 90 days.	**A)** Down-regulation of senescence markers p53, p21, p16<br>and reduction of the number of SA-β-gal-positive cells and suppression of the IL-6/STAT3 signaling pathway.<br>**B)** Increased cellular antioxidant capacity and decreased expression of SASP proinflammatory cytokines. Downregulation of COX-2, IL-1β, TNF-α, MMP-3, MMP-9 and upregulation of IL-10, TIMP-1 and SOX9 mRNA levels.<br>**C)** Improved bone mineral density, inhibition of bone resorption and reduced expression of inflammatory markers through NF-kB pathway.	**A)** [77]<br>**B)** [78]<br>**C)** [79]
	Hesperetin	**A)** Human synovial cell line SW982<br>**B)** Old wild-type (WT) mice (19.5 months to 23.5 months of age) and CISD2 reporter transgenic (TG) mice<br>**C)** Human keratinocyte cells (HEK001) from an older human subject	**A)** SW982 were treated with hesperitin (1 or 10 μM) for 24 h.<br>**B)** WT and TG mice were fed with a diet with or without hesperetin (0.07%) for 3 to 6 months.<br>**C)** HEK001 were treated with 5 μM H_2O_2 for 5 min and then with 10 μM hesperetin for 48 h. Additionally HEK001 were pre-treated with hesperetin for 2 days before exposing the cells to UVB radiation (20 J/m^2).	**A)** Inhibition of inflammatory cytokine production, JNK activity and production of MMP-3 e IL-6.<br>**B)** Extended lifespan and healthspan through activation of Cisd2.<br>**C)** Improved mitochondrial function and protected against oxidative stress via increased Cisd2 expression. Alleviated UVB-induced damage and suppressed MMP-1 expression. Activated FOXO3a and FOXM1.	**A)** [80]<br>**B)** [81]<br>**C)** [82]
	Naringenin (NAR)	**A)** 12-month-old C57BL/6 mice and mouse neural stem cell (mNSC)<br>**B)** Embryonic rat hearts (H9c2) cells<br>**C)** Human dermal fibroblasts (HDFs)<br>**D)** Nucleus pulposus cells (NPCs) from 4-week-old C57 mice<br>**E)** Embryonic rat hearts (H9c2) cells and 6-month-old C57BL/6J mice<br>**F)** *Caenorhabditis elegans* and Male 6-month-old C57BL/6J mice<br>**G)** 12-months old male WT and ApoE$^{-/-}$ C57BL/6 mice, and human aortic endothelial cells (HAECs)	**A)** NAR (20 mg/kg) was injected into caudal vein for 1 month to mice. mNSC were treated with 6.8 μg/mL of NAR for 48 h.<br>**B)** H9c2 cells were treated with H_2O_2 (5–100 μM) for 3 h and subsequently NAR (4 and 40 μM) were added to the cells and maintained in the medium for 3 days.<br>**C)** HDF cells were co-treated with NAR (5 and 10 μM) and LPS (1μM) for 24 h.<br>**D)** NPCs were exposed to IL-1β (10 and 20 ng/mL) for 48 h, then were treated with NAR (25 and 50 μM) for 48 h.<br>**E)** H9c2 cardiomyocytic cells were treated with H_2O_2 (60 μM), then with NAR (40 μM) or RES (25 μM), or sirtinol (20 μM), alone or in combination with NAR or RES for 72h. Mice were treated with NAR (100 mg/kg) diluted in water up to twelve months of age.<br>**F)** *C. elegans* were recorded from egg-lay on NGM plates containing NAR (100-400 μM for the entire lifespan. Mice were fed with NAR (100 mg/kg/day) administered in drinking water for 6 months.<br>**G)** Mice were treated with NAR (100 mg/kg/day or 200 mg/kg/day) by gavage once a day for 12 weeks. HAECs were initially treated with H_2O_2	**A)** Increased cell viability, reduced p16 expression, lengthened telomeres *in vitro* and improved neurogenesis through downregulation of TNF-α, *in vivo*.<br>**B)** Modulates oxidative stress and mitochondrial bioenergetics and mitigates H_2O_2-induced premature senescence.<br>**C)** Inhibition of NF-κB and MMPs through SIRT1 and promotes the synthesis of the ECM of cartilage, improving aging.<br>**D)** Counteracting IL-1β-induced senescence through inhibition of IGFBP-3 expression.<br>**E)** Improvement of myocardium functionality through modulation of SIRT1 and reduction of ROS production.<br>**F)** Extended lifespan and improved healthspan of *C. elegans*. Slowed down brain aging in middle-aged mice by activating SIRT1, increasing the expression of Foxo3, Nrf2, Ho-1, p16, IL-6 and IL-18.	**A)** [84]<br>**B)** [85]<br>**C)** [86]<br>**D)** [87]<br>**E)** [88]<br>**F)** [89]<br>**G)** [90]

			(60 μm) for 24 h and then with NAR (12.5, 25 and 50 μm) for 24 h.	**G)** Reduced atherosclerotic lesion formation and vascular senescence in aged $ApoE^{-/-}$ mice, decreased ROS production, and enhanced the activity and expression of SIRT-1, FOXO3a and PGC1α.	
**Flavones**	Apigenin	**A)** Male Sprague Dawley (SD) rats and human foreskin fibroblast cells (BJ)<br>**B)** Primary human primary fibroblasts (IMR-90 from fetal lung; BJ and HCA2 from neonatal foreskin) and human breast cancer cells (MDA-MB231, ZR75.1)<br>**C)** 4-month-old WT and APP/PS1 double transgenic mice<br>**D)** 6-month-old and 27-month-old male C57BL/6N mice and primary human astrocytes<br>**E)** Bone marrow-derived mesenchymal stem cells (hHBMCs)	**A)** BJ cells were treated with bleomycin (50 μg/mL) for 24 h. After the cells were washed and incubated for another 6 days. For last 24 h of treatment apigenin (10–20 μM) was added. Apigenin (2 and 4 mg/kg/day) was administered orally to 21-month-old rats for 10 days.<br>**B)** Fibroblasts were exposed to ionizing irradiation (10 Gy X-ray), and immediately given media containing apigenin and incubated. On day 9, senescent cells were given serum-free media with apigenin, and conditioned media were collected 24 h later. MDA-MB231 cells were seeded in presence of conditioned media from non-senescent or senescent fibroblasts pretreated with apigenin for 10 days.<br>**C)** WT and transgenic mice were fed with apigenin (40 mg/kg/day) dissolved in distilled water for 12 weeks.<br>**D)** Young and old mice were treated with apigenin (0.5 mg/mL) drinking water for 6 weeks. Young fetal astrocytes were subcultured for 10–12 passages to generate "aging-like" astrocytes; these cells were treated with apigenin (25 μM) for 24 h.<br>**E)** hBMSCs were exposed to osteogenic differentiation induction media supplemented with apigenin or rutaecarpine (1μM) in the presence or absence of 50 μM of the oxidative stress inducer, Tert-butyl hydroperoxide (TBHP).	**A)** Strongly inhibition of SASP, via the IRAK1/IκBα signaling pathway, *in vitro* and *in vivo*.<br>**B)** Suppression of SASP in senescent human fibroblast strains by IRAK1 and IRAK4, p38-MAPK and NF-κB. Reduction of the aggressive phenotype of human breast cancer cells.<br>**C)** Reduction of Aβ peptides accumulation, inhibit oxidative stress, and restore ERK/CREB/BDNF pathway in AD.<br>**D)** Improved learning and memory in old mice, modulated transcriptomic signatures of inflammation/immune activation and reduced markers of senescence in vitro.<br>**E)** Reduction of senescence-associated markers, SASP and ROS. Induction of HMOX1 and SOD2 expression. Enhanced osteogenic differentiation potential.	**A)** [92]<br>**B)** [93]<br>**C)** [94]<br>**D)** [95]<br>**E)** [96]

Luteolin	**A)** House Ear Institute-Organ of Corti 1 (HEI-OC1) cells<br>**B)** Immortalized human nucleus pulposus cells (HNPCs)<br>**C)** 5–6 weeks old BALB/c mice and NIH-3T3 cells.<br>**D)** 8-week-old male Wistar rats	**A)** HEI-OC1 cells were pretreated with luteolin (0.5, 1, 2, 4 μM) for 12 h, then incubated in H_2O_2 (30 μM) for 3 days.<br>**B)** HNPCs cells were treated with luteolin (1, 2, and 4 μM) or co-treated with luteolin and 50 ng/ml TNF-α for 24 h.<br>**C)** Mice's skin was pretreated with luteolin (50 nmol or 100 nmol) for 30 min, and then exposed to 10 J/cm^2 UVA. This procedure was repeated once daily for 10 consecutive days. NIH-3T3 cells were pretreated with luteolin (1, 2, 4 μM) for 30 min, and then radiated with 10 J/cm^2 UVA.<br>**D)** Rats were daily administered D-galactose (150 mg/kg/day) via subcutaneous injection once a day for 10 consecutive weeks, coupled with a single daily intraperitoneal injection of luteolin (80 mg/kg/day) starting from the seventh week and for four successive weeks.	**A)** Protects against oxidative stress-induced cellular senescence through p53 and SIRT1<br>**B)** Increased cell viability, decreased intracellular IL-1β and IL-6 expression levels and suppressed TNF-α-induced senescence via the Sirt6/NF-κB pathway.<br>**C)** Enhanced vitality and function of fibroblasts in both *in vitro* and *in vivo*. Inhibited UVA-induced collagen degradation and expression of UVA-induced senescent factors p21, p16 and SASP.<br>**D)** Alleviated D-gal-induced cognitive impairment, reversed cholinergic abnormalities and mitigated hippocampus oxidative stress, mitochondrial dysfunction, neuro-inflammation, possibly due to upregulation of SIRT1.	**A)** [98]<br>**B)** [99]<br>**C)** [100]<br>**D)** [101]
Nobiletin	**A)** Isolated human pancreatic islets from four donors<br>**B)** Male SAMP8 and SAMR1 mice<br>**C)** 9-week-old male C57BL/6 mice<br>**D)** C2C12 murine myoblasts<br>**E)** Primary human osteoarthritic (OA) chondrocytes and 10-weeks-old male C57BL/6 mice	**A)** Islets were treated with nobiletin (0.5 μM, 5 μM, and 10 μM) for 24 and 72 h.<br>**B)** Mice received intraperitoneally administration of nobiletin (10 or 50 mg/kg) daily until all behavioral experiments were completed.<br>**C)** Mice underwent hepatic ischaemia/reperfusion by clamping the hepatic artery and portal vein supplying the left and median lobes for 60 minutes, followed by reperfusion for 5 h. Nobiletin (5 mg/kg) was administered intraperitoneally at the onset of reperfusion.<br>**D)** C2C12 cells were co-treated with D-galactose (20 mg/mL) and nobiletin (10 μM) for 48 h. To obtain differentiated myotubes, C2C12 myoblasts were maintained in differentiation medium until fully differentiated (approximately 5 days) and then treated as previously described.<br>**E)** Chondrocytes were pretreated with nobiletin (10, 20, or 40 μM) for 24 h, followed by stimulation with IL-1β (10 ng/ml) for another 24 h. Mice received intraperitoneal injections of nobiletin (20 mg/kg) every two days for 8 weeks post-destabilization of the medial meniscus (DMM) surgery.	**A)** Improved islet viability, insulin and C-peptide secretion, reduced apoptosis and oxidative stress markers. Optimized skeletal muscle mitochondrial respiration and promote healthy aging.<br>**B)** Improved recognition memory, restored redox balance in the brain by normalizing the GSH/GSSG ratio and upregulating GPx and Mn-SOD enzymes.<br>**C)** Improved autophagy and mitochondrial biogenesis, through activation of the SIRT1/FOXO3a and PGC-1α pathways.<br>**D)** Enhanced mitochondrial function, reduced oxidative stress and inflammation, prevented apoptosis, activated autophagy and promoted protein homeostasis.<br>**E)** Improved synthesis of ECM proteins, inhibited cartilage degradation, reduced expression of inflammatory cytokines and prevented activation of PI3K/Akt and NF-κB.	**A)** [102]<br>**B)** [103]<br>**C)** [104]<br>**D)** [105]<br>**E)** [106]

	Tangeretin	**A)** *Caenorhabditis elegans*<br>**B)** 10–12 weeks old Swiss Wistar rats<br>**C)** Immortalized murine BV2 microglial cells and primary microglial cells from Sprague-Dawley rat pups	**A)** Worms were treated with tangeretin (30 μM and 100 μM)<br>**B)** Mice underwent transient middle cerebral artery occlusion (tMCAO) for 2 h, followed by 20 h of reperfusion to induce cerebral ischemia-reperfusion (I/R) injury. Then tangeretin (5, 10, 20 mg/kg) was administered to the rats.<br>**C)** Cells were pre-treated with tangeretin (30, 50, 100 μM) for 1 h and incubated with LPS (100 ng/ml for BV2 cells, 10 ng/ml for primary microglia) for 16 h.	**A)** Extended the mean lifespan, increased resistance to heat-shock stress, upregulation of DAF-16, HSP-16.2, and HSP-16.49, promoted the translocation of DAF-16 into the nucleus.<br>**B)** Improved neurological scores, downregulated pro-inflammatory cytokines (IL-6, IL-1β, TNF-α, IFN-γ) and upregulated TGF-β and reduced oxidative stress. Inhibition of anti-apoptotic protein Bcl-2 and Bcl-xl and modulation of PI3K/Akt signalling pathway.<br>**C)** Reduced pro-inflammatory cytokines (TNF-α, IL-6, IL-1β) and NO production, suppressed NF-κB, MAPKs, Akt pathways and activated SIRT1 and AMPK. Decreased ROS and increased HO-1 and Nrf2 activity.	**A)** [107]<br>**B)** [108]<br>**C)** [109]
**Flavonols**	Quercetin	**A)** WRN$^{-/-}$ hMSCs, LMNA$^{G608G/+}$ hMSCs and physiological-aging (PA) hMSCs<br>**B)** HFL-1 human embryonic fibroblasts<br>**C)** Human fetal lung fibroblasts (WI-38) and human osteosarcoma cell line (U2OS)<br>**D)** Male C57BL/6J mice<br>**E)** Nucleus pulposus cells (NPCs) and Sprague-Dawley rats<br>**F)** *Saccharomyces cerevisiae*<br>**G)** Human abdominal subcutaneous preadipocytes, HUVEC, primary mouse embryonic fibroblasts (MEFs), bone marrow-derived mesenchymal stem cells (BM-MSCs) and C57BL/6 mice, Ercc1−/Δ mice<br>**H)** *Caenorhabditis elegans*<br>**I)** Human umbilical vein endothelial cells (HUVECs)	**A)** All hMSCs strains were treated with quercetin (100 nmol/L).<br>**B)** Young HFL-1 cells were treated with quercetin (2 μg/ml), or quercetin caprylate (0.5, 2 and 5 μg/ml) every day until they entered senescence. Already senescent HFL-1 were treated with the same conditions.<br>**C)** WI-38 cells were treated with doxorubicin (50 nM) for 48 h and cultured in fresh complete medium for three days, then quercetin (40 μM) was added to the cultures. The conditioned media of quercetin-treated senescent WI-38 was then collected, centrifuged, and used to treat U2OS cells.<br>**D)** The mice were administered a high-fat diet for 6 month, then received treatment with quercetin (50mg/kg) 5-days biweekly via oral gavage for 10-weeks along with continued diet.<br>**E)** NPCs were treated with IL-1β then with quercetin (10 and 20 μM). A puncture-induced intervertebral disc degeneration rat model was established, followed by intragastric administration of quercetin (100 mg/kg) once daily for 4 weeks.<br>**F)** Yeast cells were pre-treated with quercetin (10 and 100 μM) for 2 h, then were exposed to oxidative stress by adding H_2O_2 (10 mM) for 1 h.<br>**G)** Preadipocytes or HUVECs and one leg of C57Bl/6 mice were irradiated with 10 Gy of ionizing radiation. Cells were then treated with dasatinib (D)(100–250 nM), quercetin (Q)(50–	**A)** Geroprotection against premature and physiological human aging.<br>**B)** Enhance lifespan and viability and shows a rejuvenating effect.<br>**C)** Reduced autophagy, increased ER stress, and partially triggered senescent fibroblast death. Reduced osteosarcoma cell invasiveness.<br>**D)** Improve obesity-related symptoms and kidney function, down regulating markers of senescence (p16, p19, p53 and SA-β-gal).<br>**E)** *In vitro* reduction of SASP and inhibition of NF-κB pathway. in IL-1β-treated cells. *In vivo* ameliorating disc degeneration.<br>**F)** Increased yeast cell survival under oxidative stress, reducing oxidative damage markers and increased longevity.<br>**G)** Combined with dasatinib, it enhances its senolytic effect *in vitro* and *in vivo* by reducing senescence markers (SA-β-Gal, p16). Delayed age-related symptoms, improved bone density, and reduced tissue pathology.<br>**H)** Extended lifespan through increased resistance to oxidative stress inhibiting SOD-3 and translocation of DAF-16 into the	**A)** [111]<br>**B)** [112]<br>**C)** [113]<br>**D)** [114]<br>**E)** [115]<br>**F)** [116]<br>**G)** [118]<br>**I)** [119]

		100 μM) or a combination of both. Mice were treated with D (5 mg/kg), Q (50 mg/kg) or D+Q.<br>**H)** Worms were pre-treated with quercetin (100 μM) for 72 h and then with 150 μM juglone.<br>**I)** HUVECs were treated with LPS (1 μg/mL), then dasatinib and quercetin (D+Q) were administered at 10 μM each for 24 h.	nucleus.<br>**I)** Inhibition of SASP via the TRAF6-MAPK-NF-κB pathway and by YTHDF2, witch bind m6A-modified MAP2K4 and MAP4K4 mRNAs, promoting their degradation and reducing the activation of the MAPK-NF-κB pathway.	
Kaempferol (KAE)	**A)** Human nucleus pulposus cells (NPCs)<br>**B)** Porcine oocytes<br>**C)** Normal human dermal fibroblasts (NHDFs), human skin equivalent (HSE) model and adult human participants.<br>**D)** *Caenorhabditis elegans*	**A)** NPCs were treated with IL-1β (10 ng/ml) for 24 h and then treated with KAE (10 μM).<br>**B)** The oocytes were cultured for 22 h without hormone to generate in vitro-aged oocytes and further cultivated in maturation medium without hormone for an additional 24 h to achieve oocytes aging. Then oocytes were exposed to KAE (0.1 μM) during *in vitro* aging.<br>**C)** NHDFs were treated with doxorubicin (100 ng/ml) and IGF-1 (100 ng/ml) for 7 days and then with KAE (2 μM). HSEs were prepared using these NHDFs, mixing a type I collagen matrix with NHDFs in a medium cocktail and incubated for 2 h, then KAE (2 μM) was applied to the HSE model. Participants at the clinical study applied a 0.1% camellia flavonoid (CF) on the right or left periorbital area and vehicle (base formulation without CF) on the other side of the face twice daily for 12 weeks. Clinical evaluations were performed at baseline and at weeks 4, 8 and 12.<br>**D)** C. *elegans* were pre-treated with KAE or fisetin (100 μM) for 48h and then exposed to thermal stress (37°C).	**A)** Attenuation of ROS levels and apoptosis rates, promoting ECM synthesis and inhibiting its degradation.<br>**B)** Increased blastocyst production rate, increased Oct4, NANOG and ITGA5 mRNA levels, reduced ROS levels, maintained mitochondrial membrane potential.<br>**C)** Inhibition of PDK1 enzyme activity, leading to downstream effects on the AKT/NFκB signaling pathway. Significant decrease in senescence markers and increase in collagen fiber content in NHDFs and HSE models. *In vivo* improved skin tissue structure and extracellular matrix composition.<br>**D)** Increased survival rates under lethal thermal stress conditions, decreased intracellular ROS levels, reduced the accumulation of lipofuscin and induced the translocation of the DAF-16 to the nucleus.	**A)** [120]<br>**B)** [121]<br>**C)** [122]<br>**D)** [123]

Rutin	**A)** Primary normal human prostate stromal cells (PSC27), breast stromal cells (HBF1203), human fetal lung stromal cells (WI38 and HFL1), human foreskin stromal cells (BJ), prostate cancer epithelial cell lines (PC3, DU145, LNCaP, M12), breast cancer epithelial cell lines (MDA-MB-231, SUM159, T47D, MCF-7). 6-weeks old NOD/SCID and C57BL/6J mice.<br>**B)** Male ApoE knockout (C57BL/6 back group) mice and primary vascular smooth muscle cells (VSMCs).<br>**C)** *Caenorhabditis elegans* and 8-weeks-old C57BL/6 mice.	**A)** Stromal cells were treated with bleomycin (BLEO) (50 μg/mL) for 7–10 days. Next 37 natural medicinal agents were tested on cells each at 3 μg/mL for 3 days, then rutin was used in the range of 20 μM to 100 μM. Stromal cells (PSC27 or HBF1203) were mixed with cancer cells (PC3 or MDA-MB-231) at a ratio of 1:4 and implanted into the hind flank of NOD/SCID mice. mitoxantrone (0.2 mg/kg doses), doxorubicin (1.0 mg/kg doses), rutin (10.0 mg/kg doses, 200 μL/dose), or vehicle controls was administered by intraperitoneal injection on the 1st day of 3rd, 5th, and 7th weeks, respectively.<br>**B)** After 6 weeks on a high-fat diet, mice received a low dose of streptozotocin (STZ, 75 mg/kg). Next rutin (40 mg/kg/day) was administered by gavage after 8 weeks of STZ administration. VSMCs were treated with H_2O_2 (10 μM) for 72h and then with rutin (50 μM).<br>**C)** Worms at larval stages L4 were treated with rutin (12.5, 25, 50 μg/mL) in nematode growth medium (NGM), and were transferred to new seeded NGM plates at days 3, 5, and 7, until the end of the reproductive period. Mice received a gavage of rutin once a day (200 mg/kg) and D-Gal was subcutaneously injected into the backs, for 7 consecutive weeks.	**A)** Limits the acute stress-associated phenotype (ASAP), interfering with ATM/HIF1α and ATM/TRAF6. Reduces pro-tumorigenic effects. In combination with chemotherapics leads to greater tumor regression increasing tumor apoptosis and reducing SASP.<br>**B)** Improved glucose and lipid metabolic disturbances in diabetic mice. Reducing atherosclerotic burden and increase in the proportion of VSMCs within the aortic root plaque. *In vitro* mitigated H_2O_2-induced premature senescence in VSMCs, through inhibition of oxidative stress and preservation of telomere integrity.<br>**C)** Increased survival rate, lifespan and reproductive output in *C. elegans*. In mice improve the exercise capacity and reduce brain tissue ROS and malondialdehyde	**A)** [125]<br>**B)** [126]<br>**C)** [127]
Fisetin	**A)** Human umbilical vein endothelial cells (HUVECs), human lung fibroblast cell line (IMR90) and primary human preadipocytes from lean male kidney donors.<br>**B)** Human umbilical vein endothelial cells (HUVECs), human aortic endothelial cells (HAECs), 27-months-old C57BL/6N mice and p16-3MR transgenic mice.<br>**C)** 22–24-month-old f1 C57BL/6:FVB and C57BL/6, $Ercc1^{-/\Delta}$ and INK-ATTAC mice. Murine embryonic fibroblasts (MEFs) from $Ercc1^{-/-}$ mice, human IMR90 fibroblasts and human adipose tissue explants from donors.<br>**D)** Female sheep aged 6–7 years.<br>**E)** Human primary adipose-derived stem cells (ADSCs) from four donors aged between 10 and 80 years.	**A)** Cells were exposed to 10 Gy ionizing radiation and then to fisetin (0.5–10 μM), and A1331852 and A1155463 (1 nM), selective BCL-XL inhibitors.<br>**B)** HUVECs and HAECs undergoing to replicative exhaustion (passage 15), then were treated with fisetin (1 μM) for 48 h. Fisetin (100 mg/kg/day) was administered via oral gavage to mice using an intermittent dosing regimen: 1 week of daily dosing, 2 weeks of no intervention, and then another 1 week of active dosing.<br>**C)** MEFs were passaged 5 times at 20% O_2. IMR90 were treated with etoposide (20 μM) for 24 h. Next both cell lines were treated with different concentrations of fisetin (1–15 μM) for 48 h. Mice received fisetin either through diet supplementation 500 ppm (500 mg/kg) or oral gavage (100 mg/kg/day).<br>**D)** Sheep received intravenous injections of fisetin at a dosage of 100 mg/kg on two consecutive days each week for a duration of 8 weeks.<br>**E)** Cells passaged up to 18 times then treated with fisetin (25 μM, 50μM, or 100 μM) for 24 h.	**A)** Induction of apoptosis in senescent HUVECs.<br>**B)** Decreased markers of cellular senescence *in vitro* and *in vivo*. Improved arterial function mediated by increasing NO bioavailability and reducing ROS.<br>**C)** Reduced number of senescent cells in both murine and human cell cultures. *In vivo* decreased senescence markers (SASP and p16) across multiple tissues, extended median and maximum lifespan, restored tissue homeostasis and alleviated age-related pathologies.<br>**D)** Reduced the number of SA-β-Gal-positive cells in the white and grey matter of the cerebral cortex. Decreased GL13-positive large neurons in the non-Cornu Ammonis area. Downregulation of senescence markers GLB1, p21 and p53 and inflammasome components (NLRP3 and TREM2).<br>**E)** Reduced ROS-positive, SA-β-gal	**A)** [15]<br>**B)** [129]<br>**C)** [130]<br>**D)** [131]<br>**E)** [132]

				and senescence-associated heterochromatin foci-positive cells while preserving differentiation potential.	
**Tannins**	Procyanidin C1 (PCC1)	**A)** Human primary prostate stromal cells (PSC27), human fetal lung fibroblasts (WI38), human umbilical vein endothelial cells (HUVECs), mesenchymal stem cells (MSCs), C57BL/6J and NOD–SCID mice	**A)** Cell lines were treated with bleomycin (50 μg/ml) for 12 h, next were maintained for 7–10 days in medium, or passaged consecutively to induce replicative exhaustion. After cells were treated with grape seed extract (GSE) (containing PCC1) at different concentrations (0.3750, 0.7500, 1.8750, 3.7500, 7.5000 and 15.0000 μg/ml). PCC1 (20 mg/kg) was administered to mice intraperitoneally in various regimens, including weekly or biweekly injections, depending on the experimental design.	**A)** *In vitro* inhibited SASP expression at low concentrations and killed senescent cells at higher concentrations, by activating intrinsic apoptotic pathways. *In vivo* extended healthspan, and amelioration of age-associated physiological decline. In combination with chemotherapics enhanced tumor regression.	**A)** [133]
**Other polyphenols**	Gingerenone A	**A)** WI-38 human diploid fibroblasts (HDFs)	**A)** Cells were exposed to 10 Gy of ionizing radiation, and then treated with gingerenone A (20 μM) for 24 or 48 h.	**A)** Induced apoptosis in senescent cells, increasing cleaved caspase-3 levels and decreasing Bcl-XL expression, reduced secretion of pro-inflammatory cytokines IL-6 and CCL2, and increasing anti-inflammatory cytokines IL-10 and IL-13.	**A)** [134]
	Hydroxytyrosol (HT) and oleuropein aglycone (OLE)	**A)** Human dermal fibroblasts (HDFs)<br>**B)** Human fetal lung fibroblasts (MRC5) and neonatal human dermal fibroblasts (NHDF).<br>**C)** C57Bl/6J mice, senescence-accelerated mouse-prone 8 (SAMP8), human fetal lung fibroblasts (MRC5) and neonatal human dermal fibroblasts (NHDF).	**A)** HDFs were pre-treated with HT (5, 10 μM) for 6 h next were exposed to UVA radiation (8 J/cm²).<br>**B)** The experiments were conducted starting from pre-senescent fibroblasts (population doubling level, PDL = 30 for MRC5 and 24 for NHDF), and terminated when the cells were either fully senescent (PDL = 40 for MRC5) or approaching senescence but still cycling (PDL 35 for NHDF). Cells were treated, from the beginning of the experiment to senescence, with OLE (10 μM) or HT (1 μM), for 4-6 weeks.	**A)** Reduced of SA-β-Gal, downregulated MMP-1 and MMP-3 and decreased IL-1β, IL-1 and IL-8 levels.<br>**B)** Decreased in SA-β-gal-positive cells, p16, COX-2, NFκB protein levels and MMP-2, MMP-9 activity.<br>**C)** *In vivo* improved contextual memory and motor coordination decreased lipid peroxidation and upregulated genes associated with synaptic plasticity and neuronal	**A)** [135]<br>**B)** [136]<br>**C)** [137]

		**C)** Mice were administered with diets enriched with high or low phenolic content extra-virgin olive oil. Fibroblast cultures were treated with HT (1 μM) or OLE (10 μM) for 4–6 weeks.	function. *In vitro* decreased the expression of inflammatory markers and improved proteasome activity.	
Curcumin	**A)** *Drosophila melanogaster*<br>**B)** *Caenorhabditis elegans*<br>**C)** 10-weeks-old male Wistar rats<br>**D)** Healthy adults aged between 60 and 85 years.	**A)** Canton-S strain received curcumin (100 μM to 250 μM) dissolved and mixed into the fly food. Ives strain received curcumin (100 μM to 250 μM) powder mixed with a yeast solution and added atop the standard food in vials.<br>**B)** Worms were exposed to curcumin (20 μM) for 72 h.<br>**C)** Rats received intraperitoneal injections of curcumin (50 mg/kg or 100 mg/kg) once a day for 28 days. These mice undergoing endurance training.<br>**D)** Participants at clinical randomized, double-blind, placebo-controlled trial received a single dose of solid lipid curcumin formulation (400 mg). Effects were evaluated at 1 and 3 hs after a single dose (acute effects), 4 weeks of daily supplementation (chronic effects), 1 and 3 hs after a single dose following chronic treatment (Acute-on-Chronic Effects).	**A)** Increased in lifespan and locomotor function, and modulated expression of several aging-related genes, including mth, thor, InR, and JNK.<br>**B)** Extended lifespan through reduction of intracellular ROS and lipofuscin and increased survival under oxidative and thermal stress.<br>**C)** Increased mitochondrial biogenesis, enhanced AMPK phosphorylation in skeletal muscle and increased SIRT1 expression.<br>**D)** Improved learning and memory abilities.	**A)** [138]<br>**B)** [139]<br>**C)** [140]<br>**D)** [141]
